# The mechanism of how CD95/Fas activates the Type I IFN/STAT1 axis, driving cancer stemness in breast cancer

**DOI:** 10.1038/s41598-020-58211-3

**Published:** 2020-01-28

**Authors:** Abdul S. Qadir, Austin M. Stults, Andrea E. Murmann, Marcus E. Peter

**Affiliations:** 1 Department of Medicine, Division Hematology/Oncology, Feinberg School of Medicine, Northwestern University, Chicago, IL 60611 USA; 2 Department of Biochemistry and Molecular Genetics, Feinberg School of Medicine, Northwestern University, Chicago, IL 60611 USA; 3 Present Address: Department of Pharmacology, College of Medicine, University of Illinois at Chicago, Chicago, IL 60612 USA

**Keywords:** Biochemistry, Cell death and immune response

## Abstract

CD95/Fas is an apoptosis inducing death receptor. However, it also has multiple nonapoptotic activities that are tumorigenic. Chronic stimulation of CD95 on breast cancer cells can increase their cancer initiating capacity through activation of a type I interferon (IFN-I)/STAT1 pathway when caspases are inhibited. We now show that this activity relies on the canonical components of the CD95 death-inducing signaling complex, FADD and caspase-8, and on the activation of NF-κB. We identified caspase-2 as the antagonistic caspase that downregulates IFN-I production. Once produced, IFN-Is bind to their receptors activating both STAT1 and STAT2 resulting in upregulation of the double stranded (ds)RNA sensor proteins RIG-I and MDA5, and a release of a subset of endogenous retroviruses. Thus, CD95 is part of a complex cell autonomous regulatory network that involves activation of innate immune components that drive cancer stemness and contribute to therapy resistance.

## Introduction

CD95/Fas is a well characterized death receptor that, in permissive cells, mediates induction of apoptosis when stimulated by its cognate ligand CD95L^1–3^. Apoptosis induction involves activation of a caspase cascade that initiates with the formation of the death inducing signaling complex (DISC) comprising the adaptor protein FADD and caspase-8^4^. However, it is now well established that CD95 also has multiple cancer relevant nonapoptotic functions^5,6^. One of the many reported nonapoptotic activities of CD95 is to drive and maintain cancer stem cells (CSCs)^7–9^.

Type I interferons (IFN-I) play essential roles in establishing and modulating host defense against microbial infection via induction of IFN-stimulated genes (ISGs) through Janus kinases (JAKs) and signal transducers and activators of transcription (STATs). STAT1 and STAT2 are key regulators of the IFN-I pathway^10^. One way they are activated is after binding of IFN-I (IFNα and IFNβ) to the IFN-I receptors, IFNAR1 and IFNAR2. This results in phosphorylation of STAT1/2 by JAK1, JAK2 and TYK2. In the canonical antiviral immune response tyrosine-phosphorylated STAT1/2 heterodimers bind to IRF9, forming IFN-stimulated gene factor 3 (ISGF3), which binds to IFN-stimulated response elements (ISREs) in the promoters of ISGs to initiate their transcription^11^.

IFN-Is are induced in response to a variety of viral double stranded (ds)RNAs that either bind to surface receptors (e.g. Toll-like receptors 3, 7 and 8)^12^ or to soluble cytosolic sensors^13^. Retinoic acid-inducible gene I (RIG-I) and melanoma differentiation-associated antigen 5 (MDA5) are soluble receptors that sense dsRNAs of different lengths^14^. They both interact with the mitochondrial signaling adaptor MAVS. Its oligomerization results in activation of the transcription factors IRF3/7 and NF-κB^15^. While the RNA sensors are part of a host defense system against viral infections, the boundaries between self and non-self have become blurred by reports demonstrating that endogenous retroelements such as endogenous retroviruses (ERVs) can trigger an innate immune response through activating dsRNA sensors^13^. It was demonstrated that ERVs could be mobilized by treating cancer cells with DNA methyltransferase (DNMT) inhibitors resulting in the activation of the dsRNA sensors MDA5 and TLR3^16,17^.

This viral RNA sensing mechanism is often viewed as antiproliferative and in many cases to induce apoptosis or other forms of cell death^18^. Similarly, in the context of cancer, activation of STAT1 and its target genes are viewed as a tumor suppressive^19,20^. However, a growing number of reports suggest that STAT1 could have tumor promoting activities^21–24^. STAT1 in particular has also been linked to therapy resistance^25–28^. We recently reported that long-term stimulation of CD95 results in induction of IFN-I expression. The activation of IFNAR1/2 in turn activates STAT1 and ISGs driving cancer stemness^29^. We showed IFN-Is, activation of IFNARs, and of JAK kinases followed by activation of STAT1 were critical for this activity. In the context of breast cancer we found that treating cancer cells directly with IFN-I also increased their stemness and the frequency of cancer initiating cells (CICs) *in vivo*.

We now demonstrate that CD95 signaling promotes STAT1 induction either through caspase 8 activation in which caspase-8 catalytic activity is required, and caspase-2 is antagonistic, as well as activation of NF-κB, or through activation of dsRNA sensing pathways, which respond to an upregulation of ERVs by promoting STAT1 signaling. Furthermore, we show that downstream signaling through STAT1 that drives cancer stemness is dependent on STAT2, but is inhibited by STAT3. In summary, CD95 is part and a complex regulatory network that in apoptosis resistant cancer cells can drive increased stemness and therapy resistance.

## Results

### The CD95 DISC components FADD and caspase-8 are required for CD95 to activate STAT1

**Figure 1:**
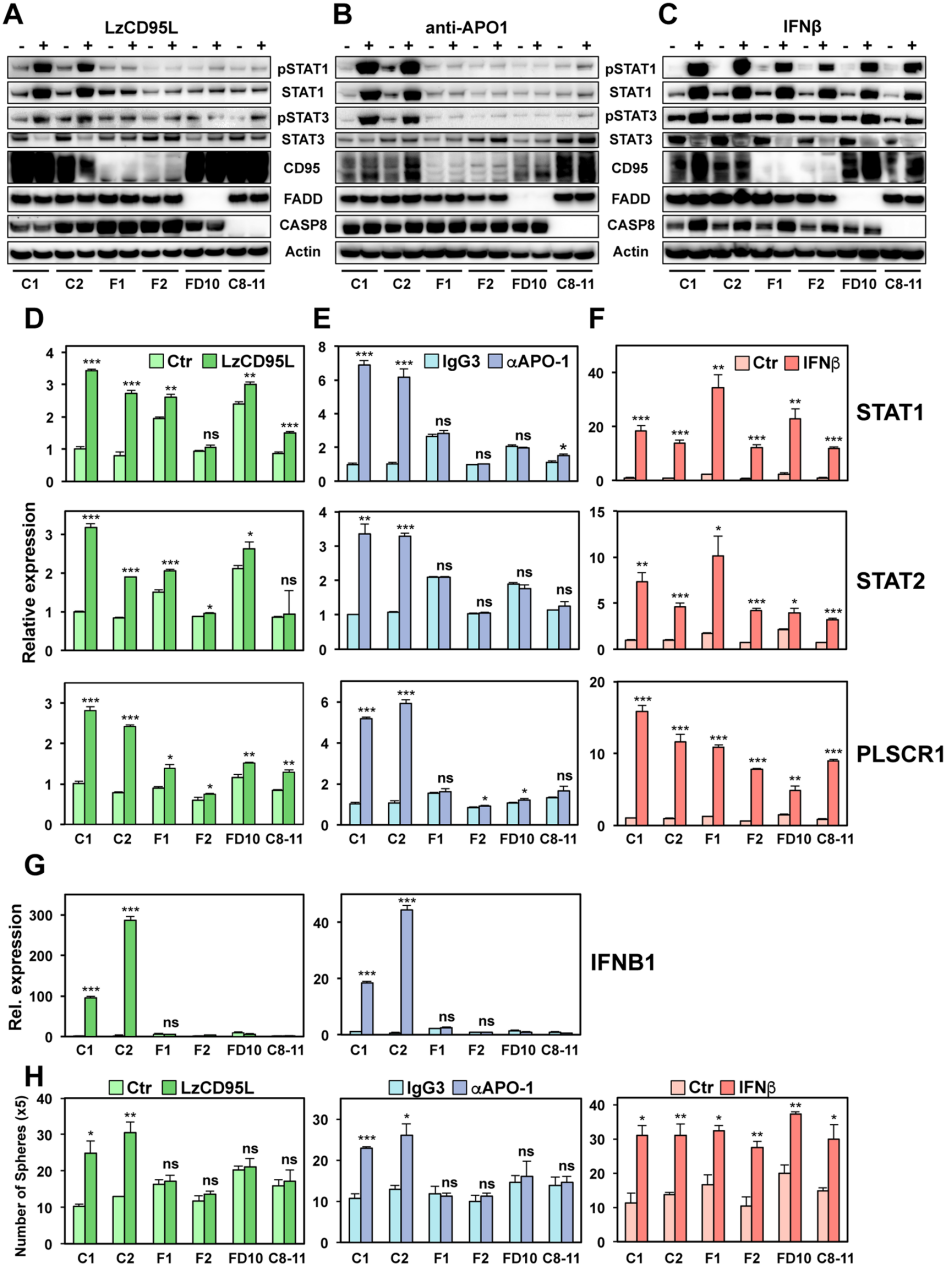
DISC components, CD95, FADD and caspase-8 are required for STAT1 phosphorylation and IFN-I expression upon prolong stimulation of CD95. (**A**–**C**) Western blot analysis of Cas9 transfected wt clones (C1 and C2) and CD95 (F1-complete CD95 k.o., F2-CD95 exon 4 deletion), FADD (FD10) and caspase-8 (C8–11) k.o. clones of MCF-7 either control treated or treated with LzCD95L (**A**), anti-APO-1 (**B**) or IFNβ (**C**) for 4 days. All uncropped immunoblot images are included in Fig. S9. (**D–F**) Real-time PCR quantification of mRNAs in wt clones or CD95, FADD, and caspase-8 k.o. clones of MCF-7 treated with either LzCD95L (**D**), anti-APO-1 (**E**) or IFNβ (**F**) for 4 days. (**H**) Number of single cell spheres formed after 6 days by Cas9 transfected wt clones or CD95, FADD, or caspase-8 k.o. clones of MCF-7 plated in 24 well plates treated with either LzCD95L or anti-APO-1 or IFNβ for 6 days. Error bars represent the SD of three biological replicates. Student’s *t-*test was performed compared to matching control. p-value *<0.05, **<0.001; ***<0.0001; ns, not significant.

We previously showed that in a number of CD95 expressing breast cancer cells and in a squamous carcinoma cell line, prolonged stimulation through CD95 caused upregulation of IFN-I. These upregulated type I IFNs, through binding to their receptors, activated STAT1 driving the transcriptional upregulation of ISGs. Activation of this signaling axis resulted in an increase in cancer stemness^29^. While we reported that one or more caspases inhibited this signaling pathway, the signaling components downstream of CD95 remained unknown. To study the signaling downstream of CD95 that engages this pathway, we generated CD95, FADD and caspase-8 k.o. MCF-7 cells (Fig. S1A,B, and^30^) to determine whether the key components of the CD95 DISC are required for this activity of the receptor. STAT1 phosphorylation was monitored as a marker of activation of the CD95/IFN-I/STAT1 pathway. As expected, the knock-out of CD95 completely prevented the activation of STAT1 in cells stimulated either with soluble CD95L (LzCD95L) (Fig. 1A and Fig. S1C, left panel) or with the agonistic mAb anti-APO-1 (Fig. 1B). This confirmed that the strong innate immune response seen in these cells was solely caused by stimulation of the CD95 receptor. The lack of CD95 expression had no effect on the activation of STAT1 in cells treated with IFNβ (Fig. 1C and Fig. S1C, right panel), which by-passes the requirement for CD95 stimulation, confirming that IFNR signaling to STAT1 is downstream of CD95. Consistent with canonical CD95 signaling being required for engaging this nonapoptotic pathway, knock-out of either FADD or caspase-8 also completely blocked activation of STAT1 in cells treated with either CD95L or anti-APO-1 (Fig. 1A,B, and Fig. S1D,E, left panels). As with CD95 k.o., activation of STAT1 in the mutant cells treated with IFNβ was not affected (Fig. 1C and Fig. S1D,E, right panels). Activation of the STAT1 mediated signaling pathway resulting in the induction of STAT1 regulated genes was confirmed by real time (q)PCR of STAT1, STAT2 and of the most strongly induced STAT1 regulated gene we had identified downstream in this pathway, PLSCR1^29^ (Fig. 1D–F). Induction of these genes after CD95 stimulation was strongly reduced in the three knock-out cells (Fig. 1D,E) while there was no impairment when the cells were treated directly with IFNβ (Fig. 1F). Consistently, only the wild-type (wt) cells were able to induce expression of IFNβ when stimulated through CD95 (Fig. 1G). In agreement with our previous results, activation of STAT1 increased the propensity for the wt cells to form spheres (Fig. 1H), downregulate CD24 (Fig. S2A) and increase their ALDH1 activity (Fig. S2B). All these signs of increased stemness were blunted in the k.o. cells. In summary, the data suggest that the increased cancer stemness seen in cells stimulated through CD95 is dependent on the expression of the two main DISC components FADD and caspase-8.

### Involvement of caspases in regulating CD95 mediated STAT1 activation

**Figure 2:**
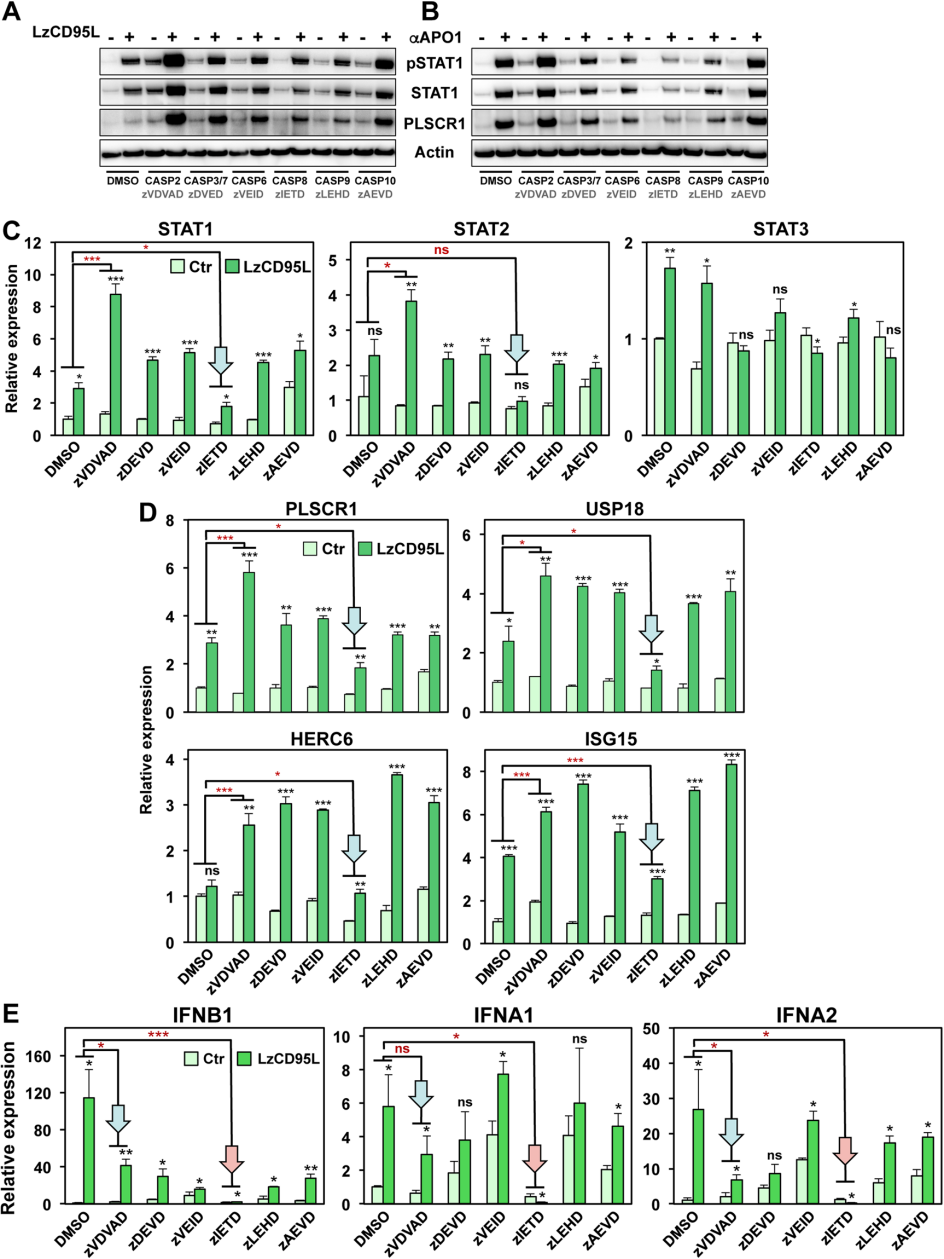
Effect of different caspase inhibitors on STAT1 activation and STAT1 target gene expression in CD95 stimulated cells. (**A**,**B**) Western blot analysis of MCF-7 cells treated with either DMSO solvent control, 20 μM of an inhibitor of caspase-2 (zVDVAD), caspase-3/7 (zDEVD), caspase-6 (zVEID), caspase-8 (zIETD), caspase-9 (zLEHD), or caspase-10 (zAEVD) upon LzCD95L (**A**) or anti-APO-1 (**B**) treatment for 4 days. All uncropped immunoblot images are included in Fig. S9. (**C**) Real-time PCR quantification of mRNAs in MCF-7 cells treated as shown in A upon exposure to LzCD95L for 4 day. (**D**) Real-time PCR quantification of mRNAs in MCF-7 cells treated as in A. Error bars represent the SD of three biological replicates. (**E**) Real-time PCR quantification of mRNAs in MCF-7 cells treated as in A. Student’s *t-*test was performed compared to matching control. A linear model for continuous gene expression levels, using binary predictors for LzCD95L and zVDVAD or zIETD and their interaction term, was used to evaluate whether the effect of LzCD95L on gene expression varied depending on the presence of zVDVAD or zIETD (red asterisks). p-value *<0.05, **<0.001; ***<0.0001; ns, not significant.

We previously showed that one or more caspases were negative regulators of the CD95/IFN-I/STAT1 signaling axis^29^. Inhibition of caspases using the oligo caspase inhibitor zVAD-fmk (zVAD) in multiple of cancer cell lines enhanced or even derepressed the activation of STAT1 in CD95 stimulated cells. To assess which caspase(s) might be inhibiting signaling to STAT1, we treated MCF-7 cells with various caspase inhibitors while stimulating them through CD95 (Fig. 2). Only the caspase-8 inhibitor zIETD consistently reduced STAT1 phosphorylation, STAT1 and STAT2 upregulation, and the expression of the main STAT1 targets at the protein level (Fig. 2A,B and Fig. S3A, left and center panel) and mRNA level (arrows in Fig. 2C,D). This result suggests that the enzymatic activity of caspase-8 was required for CD95 to engage the STAT1 pathway. Consistently, we could not correct the defect in the caspase-8 k.o. cells by overexpressing a catalytically inactive caspase-8 gene (Fig. S3B,C).

Of all tested inhibitors, the caspase-2 inhibitor zVDVAD was most active in increasing phosphorylation of STAT1 and the expression of STAT1 and PLSCR1 on the protein level in CD95 stimulated cells (Fig. 2A,B). On the mRNA level this was also seen for PLSCR1. The activation of three other genes that we recently identified by ChIP-Seq as being directly regulated by STAT1 in CD95 stimulated cells, was also examined. These genes, USP18, HERC6 and ISG15^29^, were equally increased by the treatment with other caspase inhibitors as well as caspase-2 inhibitor, except when treated with the caspase-8 inhibitor zIETD, which was inhibiting (Fig. 2D). Overall the caspase-2 inhibitor seemed to be most effective in driving STAT1 pathway activation in CD95 stimulated cells. Interestingly, zVDVAD is structurally related to zVAD which we had shown before to increase STAT1 activation upon CD95 stimulation^29^ pointing at caspase-2 as a likely candidate to negatively regulate CD95 mediated STAT1 activation. In contrast, the caspase-3/7 inhibitor zDEVD did not enhance STAT1 activation (Fig. 2A,B and Fig. S3A, right panel). To test the contribution of caspase-2 to the activation of STAT pathways through CD95, we knocked out caspase-2 using CRISPRi (Fig. S4A). In the majority of cells, absence of caspase-2 resulted in the activation and upregulation of STAT1 as well as its target gene, PLSCR1, even without CD95 stimulation (Fig. S4B). This was also observed at the RNA level (Fig. S4C). We noticed that caspase-2 protein levels in wt cells were somewhat reduced upon CD95 stimulation (Fig. S4B) suggesting that part of the activation of STAT1 downstream of CD95 may be due to the reduction of the negative regulator caspase-2. Based on the observation that caspase-2 inhibition does not block but rather enhances this signaling, it is not likely that processing/activation of caspase-2 is required for activation of STAT1.

An apparent contradiction in our data was found in the effect the inhibitors zVDVAD and zVAD on one hand, and the caspase-8 inhibitor zIETD on the other hand, had on activation of STAT1 in CD95 stimulated cells: zVDVAD/zVAD consistently promoted this activation whereas zIETD blocked it. Given the fact that zVAD inhibits both caspase-2 and caspase-8^31^ this is difficult to reconcile. To determine whether the enzyme inhibited by zVAD was upstream or downstream of caspase-8 in the pathway we pretreated caspase-8 k.o. cells with zVAD and stimulated them with LzCD95L (Fig. S3D). This did not allow the cells to activate STAT1 suggesting that the enzyme inhibited by zVAD is downstream of the active caspase-8 produced by the DISC. While caspase-2 inhibition or deletion enhances activation of STAT1 and its target genes (see Fig. 2B,C, Fig. S4B,C), paradoxically it resulted in a reduced ability of the CD95 stimulated cells to upregulate IFN-I (blue arrows in Fig. 2E). It did not shut it down as inhibition caspase-8 did (red arrows in Fig. 2E) but it dampened it. In summary, the data suggest that caspase-8 promotes and caspase-2 inhibits the nonapoptotic activity of CD95 resulting in the production of IFN-I. Further, caspases may be involved in regulating this activity both upstream and downstream of the activation of STAT1.

### Mitochondrial function and canonical NF-κB activation are required for CD95 to activate IFN-I

**Figure 3:**
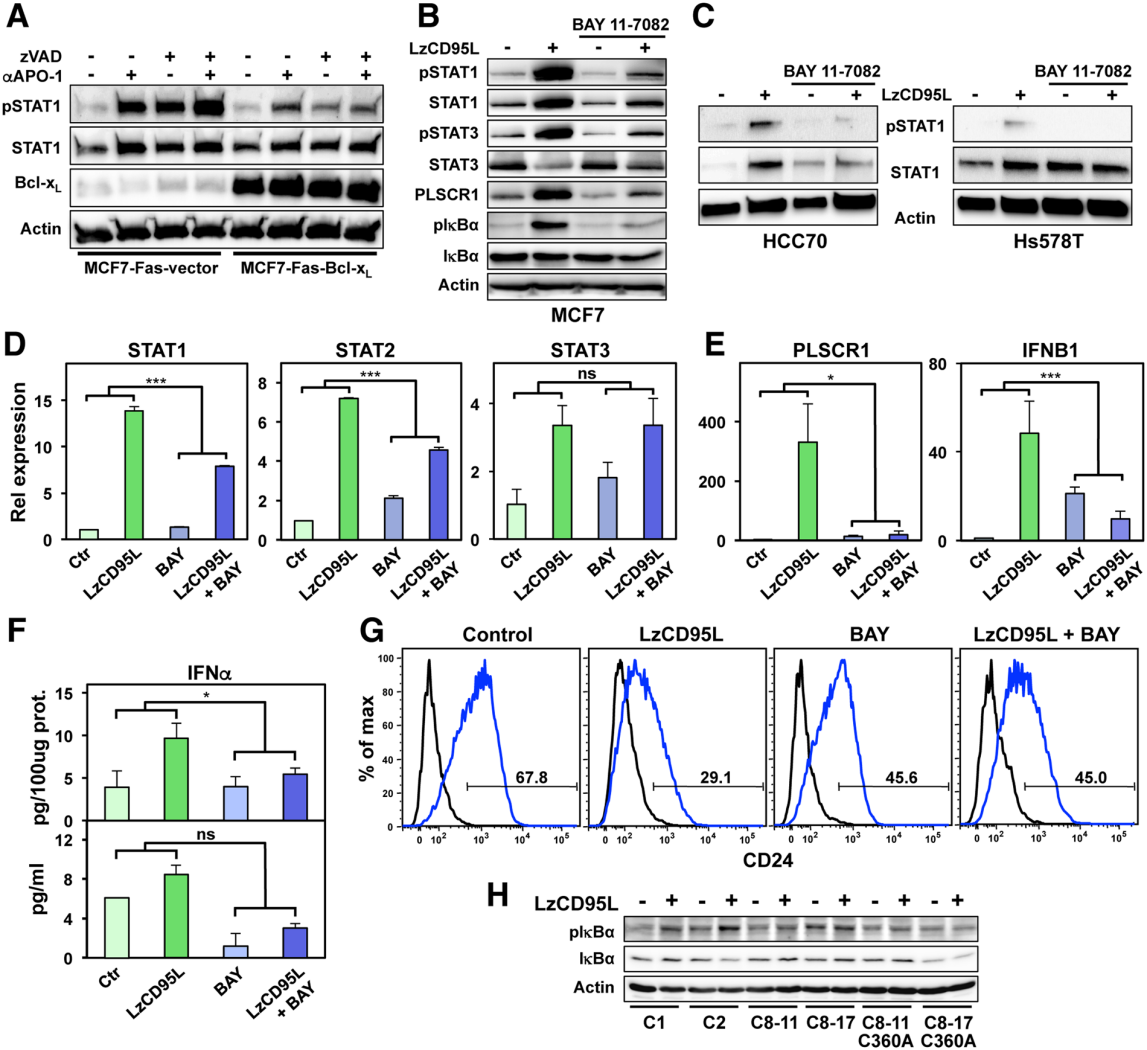
NF-κB activation is involved in IFN-I induction upon CD95 stimulation. (**A**) Western blot analysis of MCF-7-Fas-vector or MCF-7-Fas-Bcl-x_L_ cells either control IgG3 treated (−) or treated with anti-APO-1 (+) in the presence of absence of 20 μM zVAD for 4 days. (**B**) Western blot analysis of MCF-7 cells treated with either solvent control or with 5 μM of BAY 11–7082 left untreated or stimulated with LzCD95L for 4 days. (**C**) Western blot analysis of HCC70 (left panel) or Hs578T (right panel) breast cancer cells treated with either solvent control or with 5 μM of BAY 11–7082 left untreated or stimulated with LzCD95L for 4 days. All uncropped immunoblot images are included in Fig. S9. (**D**,**E**) Real-time PCR quantification of mRNAs in MCF-7 cells treated with either solvent control or with 5 μM of BAY 11–7082 left untreated or exposed to LzCD95L for 4 day. (**F**) ELISA quantification of IFNα protein produced by MCF-7 cells left untreated or treated with LzCD95L in the presence of solvent control or 5 μM of BAY 11–7082 for 2 days. (**G**) CD24 surface staining of MCF-7 cells left untreated or stimulated with LzCD95L treated with solvent control or 5 μM of BAY 11–7082 for 6 days. Error bars represent the SD of three biological replicates. A linear model for continuous gene expression levels, using binary predictors for LzCD95L and BAY and their interaction term, was used to evaluate whether the effect of LzCD95L on gene expression varied depending on the presence of BAY. p-value *<0.05, ***<0.0001; ns, not significant. (**H**) Western blot analysis of Cas9 control clones (C1 and C2), two caspase-8 k.o. clones, or two caspase-8 k.o. clones reconstituted with a catalytically inactive caspase-8 gene (C8–11-C360A and C8–17-C360A) after LzCD95L treatment for 4 days.

We previously demonstrated that MCF-7 cells are Type II cells, as they require mitochondria to activate the apoptosis pathway^32^. To determine whether mitochondrial function was also required for the ability of CD95 to activate the IFN-I/STAT1 pathway, we chronically treated MCF-7-Fas cells overexpressing Bcl-x_L_ with anti-APO-1 (Fig. 3A). The activation of STAT1 was severely reduced in the Bcl-x_L_ expressing cells and this could not be reversed by inhibiting caspases. This suggests that the pathway that leads to activation of STAT1 at least in MCF-7 cells relies on mitochondrial function. To determine whether canonical nonapoptotic CD95 signaling was involved in CD95 stimulating IFN signaling, we first tested whether RIP1 signaling (expression of which was strongly induced) was involved. The RIP1 kinase inhibitor Necrostatin-1 did not inhibit CD95 mediated activation of STAT1 (Fig. S5A). In contrast, treatment of cells with the NF-κB inhibitor BAY 11–7082 strongly reduced the ability of CD95 to activate STAT1 in CD95 stimulated MCF-7 cells (Fig. 3B), and two other breast cancer cell lines we recently showed activate STAT1 in response to CD95 stimulation (Fig. 3C)^29^. The ability of CD95 to activate STAT1 was also blunted in cells overexpressing a dominant negative mutant IκBα (Fig. S5B). Activation of NF-κB was required for upregulation of both STAT1 and STAT2 but not of STAT3 (Fig. 3D). Inhibition of NF-κB blocked induction of PLSCR1 and IFNB1 (Fig. 3E) and reduced the release of IFNα protein (Fig. 3F). Consequently, loss of CD24 as a measure of increased stemness was also reduced (Fig. 3G and Fig. S5C). Finally, activation of NF-κB was confirmed to occur downstream of the DISC and to require active caspase-8 (Fig. 3H).

### Chronic CD95 stimulation causes activation of IRF3/7 through dsRNA sensors

**Figure 4:**
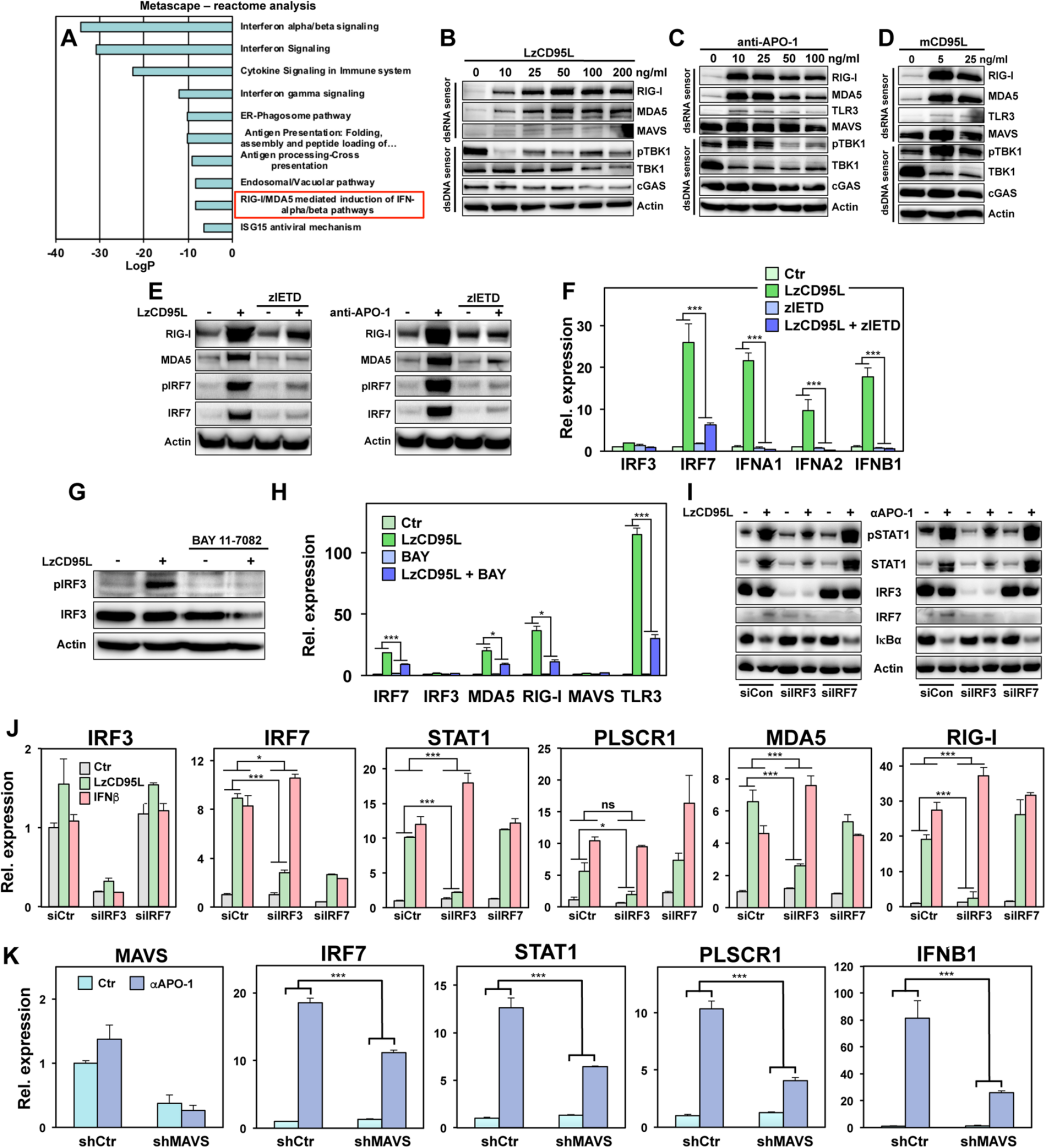
Long-term stimulation of CD95 induces upregulation of dsRNA sensor genes and activation of IRF3/7. (**A**) Metascape-reactome analysis of up- or downregulated genes detected by microarray analysis in 14 days anti-APO-1 stimulated MCF-7 cells. (**B**–**D**) Western blot analysis of MCF-7 cells treated with different concentrations of LzCD95L (**B**), anti-APO-1 (**C**), or membrane bound CD95L (**D**) for 4 days. (**E**) Western blot analysis of MCF-7 cells 4 days after stimulation with through CD95 with or without treatment with 20 μM zIETD. (**F**) Real-time PCR quantification of mRNAs in MCF-7 cells left untreated or treated with LzCD95L for 4 days in the presence or absence of 20 μM zIETD. (**G**) Western blot analysis of MCF-7 cells treated with solvent or with 5 μM of BAY 11–7082 after LzCD95L treatment for 4 days. (**H**) Real-time PCR quantification of mRNAs in MCF-7 cells in the presence of solvent control or 5 μM of BAY 11–7082 left untreated or exposed to LzCD95L for 4 days. (**I**) Western blot analysis of MCF-7 cells transfected with control siRNA (siCtr) or smart pool siRNA against IRF3 (siIRF3) or IRF7 (siIRF7) and incubated with either LzCD95L (left panel) or anti-APO-1 (right panel) for 4 days. All uncropped immunoblot images are included in Fig. S9. (**J**) Real-time PCR quantification of mRNAs in MCF-7 cells transfected with control siRNA (siCtr) or smart pool siRNA against IRF3 (siIRF3) or IRF7 (siIRF7) left untreated or incubated with LzCD95L or IFNβ for 4 days. (**K**) Real-time PCR quantification of mRNAs in MCF-7 cells infected with a control shRNA expressing lentivirus (shCtr) or with an shRNA targeting MAVS and incubated with either IgG3 (Ctr) or anti-APO-1 (αAPO-1) for 4 days. Error bars for MDA5, RIG-I, MAVS, TLR3 (**H**) and MAVS (**K**) represent the variance of two biological replicates. All other error bars represent the SD of three biological replicates. F, H, J and K: A linear model for continuous gene expression levels, using binary predictors for a stimulant (LzCD95L, anti-APO-1 or IFNβ) and an modulator (zIETD, BAY or shMAVS) and their interaction term, was used to evaluate whether the effect of the stimulant on gene expression varied depending on the presence of the modulator. p-value *<0.05, **<0.001; ***<0.0001; ns, not significant.

To determine the mechanism through which CD95 caused induction of IFN-I, we subjected our recent gene array data on CD95 stimulated MCF-7 cells^29^ to a Metascape reactome analysis. In addition to the expected GO signatures that are consistent with IFN-I activation, we found evidence of an induction of the IFN-I pathway through the double stranded (ds)RNA sensors RIG-I/MDA5 (Fig. 4A). Western blot and qPCR analyses confirmed that RIG-I, MDA5 and TLR3 but not MAVS were strongly upregulated in CD95 stimulated cells (Fig. 4B–D, Fig. S6A,B) and this upregulation could be blocked by treating cells with zIETD (Fig. 4E). RIG-I and MDA5 mRNA was also not induced in caspase-8 k.o. cells (Fig. S6C). In contrast, the dsDNA sensor cGAS was not induced at the protein level (Fig. 4B-D) and only a small increase of both cGAS and STING mRNA was detectable (Fig. S6A,B). These data suggest that the dsRNA sensors RIG-I and MDA5 are activated downstream of CD95 stimulation.

dsRNA sensors have been shown to connect to IFN-I induction via activation of IRF3 and IRF7, as well as through NF-κB^33^. Consistently, we found a strong upregulation of IRF7 protein and mRNA in CD95 stimulated cells (Fig. 4E,F, Fig. S6A,B). This was inhibited by treatment with zIETD (Fig. 4E,F) or deletion of CD95, FADD or caspase-8 in k.o. cells (Fig. S6D). IRF7 could still be induced by treating any of the k.o. cells with IFNβ suggesting that its activation was downstream of STAT1 activation.

In contrast to IRF7, IRF3 was not found to be upregulated on either the protein (Fig. 4G) or the mRNA level (Fig. 4F, and Fig. S6A), except in cells treated with a very high concentration of anti-APO-1 (Fig. S6B). IRF3 was found to be phosphorylated in stimulated cells and this phosphorylation was blocked in cells with inhibited NF-κB (Fig. 4G) as was the induction of IRF7, MDA5, RIG-I and TLR3 mRNA (Fig. 4H). These data suggested that NF-κB could be both upstream and downstream of IRF3/7. To determine the functional role of both IRF3 and IRF7 in the activation of NF-κB and the activation of STAT1 and its target genes, we knocked down either IRF3 or IRF7 in CD95 stimulated cells (Fig. 4I,J, left two panels). Knockdown of IRF7 did not have an effect on activation of NF-κB, as shown by loss of IκBα expression, and subsequent STAT1 phosphorylation in cells stimulated through CD95 (Fig. 4I). It also did not inhibit the induction of the expression of STAT1 and PLSCR1 mRNA or of MDA5 and RIG-I (Fig. 4J). In contrast, knockdown of IRF3 substantially attenuated all of these placing IRF3 upstream of these activities. Finally, we generated cells with a stable knockdown of MAVS (Fig. 4K). These knockdown cells showed a substantial reduction in their ability to upregulate IFNB1 and STAT1 upon CD95 stimulation, suggesting that activation of the dsRNA sensors contributes to the activation of the IFN-I pathway. In summary, chronic CD95 stimulation causes induction of IFN-I through a NF-κB-dependent pathway resulting in the induction of dsRNA sensors and IRF3.

### Chronic stimulation of CD95 causes the release of a subset of endogenous retroviruses

**Figure 5:**
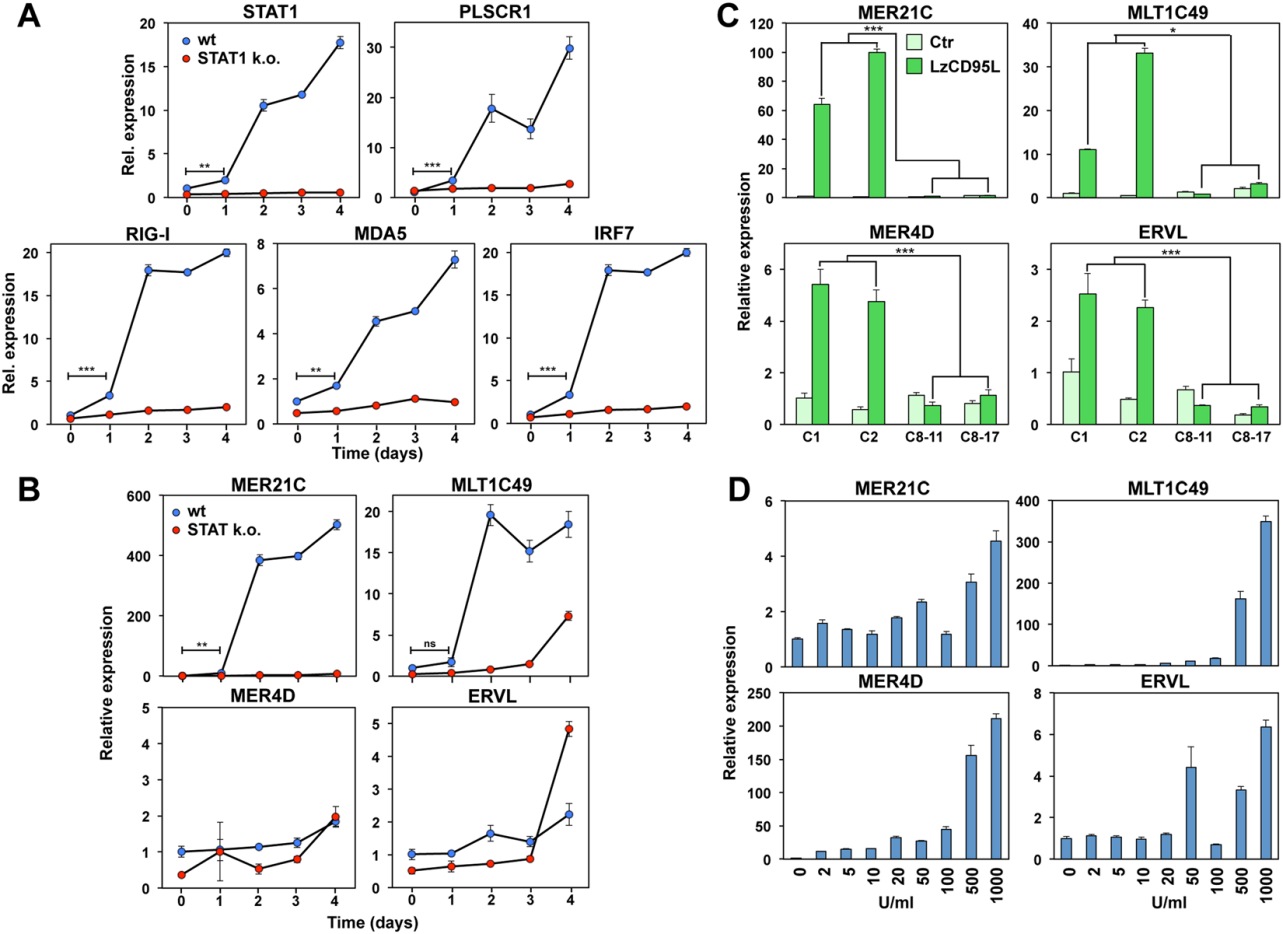
Long-term stimulation of CD95 induces upregulation of endogenous retroviruses. (**A**) Real time PCR analysis of mRNAs of STAT1, PLSCR1, RIG-I, MDA5, and IRF7 of Cas9 control (wt, clone C1) and STAT1 k.o. MCF-7 cells (clone S1–6) after 0, 1, 2, 3 or 4 days treatment with LzCD95L. Error bars represent the SD of three biological replicates. Student’s *t-*test was performed to compare gene expression between 0 and 1 days treatment in wt samples. (**B**) Real time PCR analysis of selected ERVs MER21C, MLT1C49, MER4D, and ERVL of Cas9 control (wt, clone C1) and STAT1 k.o. cells (clone S1–6) after 0, 1, 2, 3 or 4 days treatment with LzCD95L. Error bars represent the SD of three biological replicates. Student’s *t-*test was performed to compare gene expression between 0 and 1 days treatment in wt samples. (**C**) Real time PCR analysis of selected ERVs in two Cas9 control clones (C1 and C2) and two caspase-8 k.o. clones (C8–11 and C8–17) after 4 days treatment with LzCD95L. Error bars represent the SD of three biological replicates. Student’s *t-*test was performed to compare gene expression between the average of LzCD95L treated control and two caspase-8 k.o. clones. (**D**) Real time PCR analysis of selected ERVs of MCF-7 cells after 4 days or treatment with different concentrations of IFNβ. Error bars represent the SD of four biological replicates. p-value *<0.05, **<0.001; ***<0.0001; ns, not significant.

As early as one day after starting CD95 stimulation a small but significant level of STAT1, PLSCR1, RIG-I, MDA5 and IRF7 induction could be detected and their expression levels dramatically increased after two days (Fig. 5A). This cellular response was dependent on STAT1. Treatment with IFNβ also strongly induced these same genes with an IFNβ concentration as low as 2 U/ml (Fig. S7A–C). Given the fact that we found a profound and sustained upregulation of dsRNA sensors RIG-I and MDA5, we tested whether expression of their natural substrates, endogenous retroviruses are upregulated in chronically CD95 stimulated cells. We tested four ERVs strongly upregulated in cells treated with demethylating reagents^17^. This treatment was shown to activate the RIG-I/MDA5 pathway leading to activation of NF-κB through induction of IRF7 and phosphorylation of IRF3 - both events detected in CD95 stimulated cells - eventually resulting in induction of IFN-I. We detected a dramatic mobilization of two of the four tested ERVs (MER21C and MLT1C49) in response to CD95 stimulation which was strongly dependent on the presence of STAT1 (Fig. 5B). The effect was specific for certain ERVs, as MER4D was not more highly expressed in wt than STAT1 k.o. cells and ERVL showed higher and late induction in the STAT1 k.o. cells (Fig. 5B). This suggested a high complexity in the regulation of ERVs by STAT1. None of the ERVs showed an increase in expression in caspase-8 k.o. cells confirming that they are all downstream of canonical CD95 signaling (Fig. 5C). Interestingly, robust induction of ERVs upon IFNβ treatment required higher IFNβ concentrations (Fig. 5D) than required to induce the dsRNA sensor proteins (Fig. S7A–C). Upregulation of dsRNA sensors and ERVs were also found in other cell lines when stimulated through CD95 (Fig. S8) indicating that this activity of CD95 is not limited to MCF-7 cells. Our data are consistent with a model in which induction of ERVs is involved in helping sustain the innate immune-like activity in CD95 stimulated cells.

### CD95 induced activation of IFN-I pathway involves both STAT1 and STAT2

**Figure 6:**
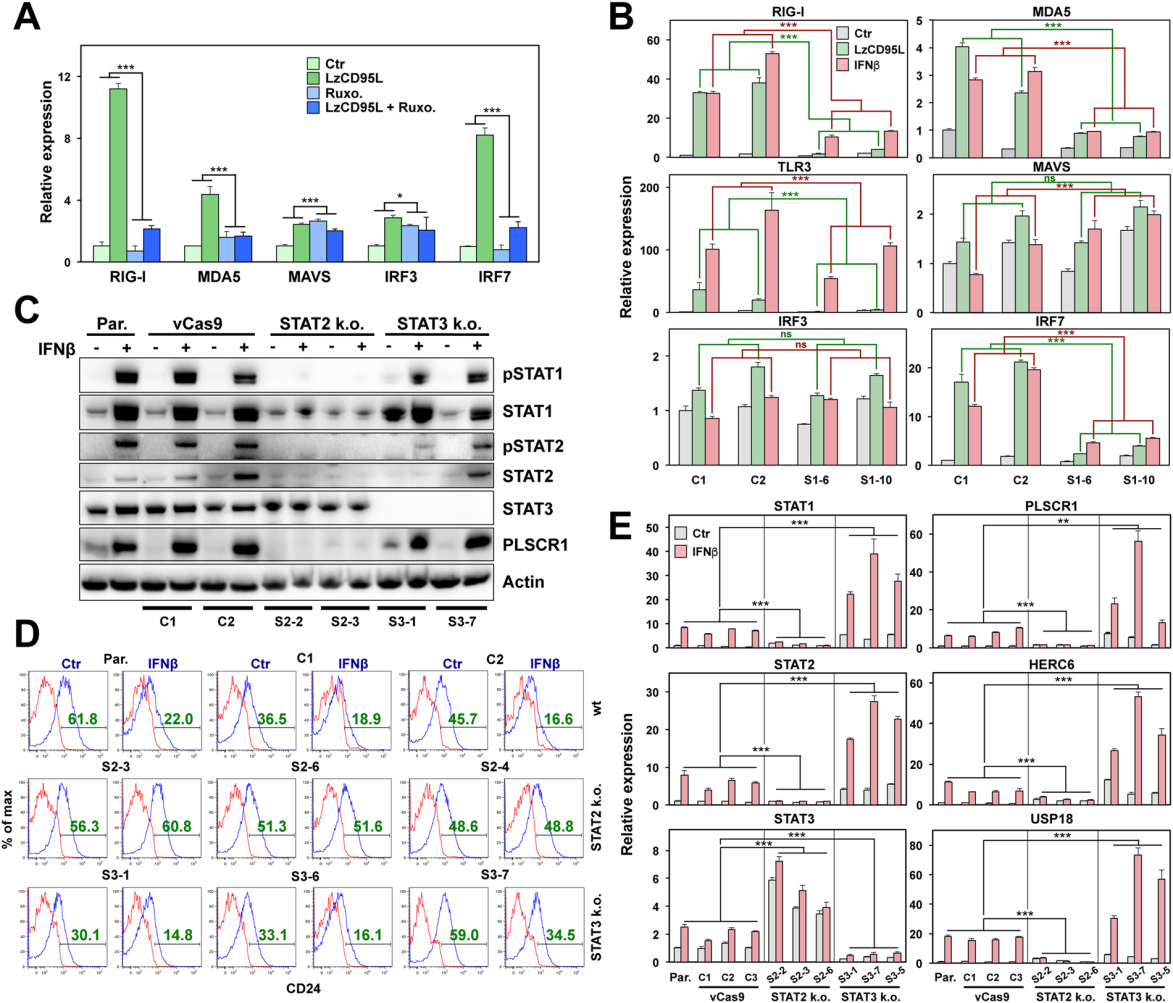
Induction of IFN-I pathway requires both STAT1 and STAT2 upon CD95 stimulation. (**A**) Real time PCR analysis of mRNAs of RIG-I, MDA5, MAVS, IRF3, and IRF7 of MCF-7 cells control treated or treated with LzCD95L for 4 days in presence of solvent control (Ctr) or Ruxolitinib (Ruxo.). Error bars represent the SD of three biological replicates. A linear model for continuous gene expression levels, using binary predictors for LzCD95L and Ruxo. and their interaction term, was used to evaluate whether the effect of LzCD95L on gene expression varied depending on the presence of Ruxo. (**B**) Real time PCR analysis of mRNAs of RIG-I, MDA5, TLR3, MAVS, IRF3, and IRF7 of two Cas9 control clones (C1 and C2) and two STAT1 k.o. clones (S1–6 and S1–10) 4 days after control treatment or treated with either LzCD95L or IFNβ. Error bars represent the SD of four biological replicates. Student’s *t-*test was performed to compare gene expression between the average of LzCD95L (green) or IFNβ (pink) treated control and two STAT1 k.o. clones. (**C**) Western blot analysis of MCF-7 cells, viral (v)Cas9 infected wt clones (C1 and C2), STAT2 k.o. clones (S2–2 and S2–3), or STAT3 k.o. clones (S3–1 and S3–7) either control treated or treated with IFNβ for 4 days. All uncropped immunoblot images are included in Fig. S9. (**D**) CD24 surface staining of MCF-7 cells, vCas9 infected wt clones (C1 and C2), STAT2 k.o. clones (S2–3, S2–6 and S2–4), or STAT3 k.o. clones (S3–1, S3–6 and S3–7) either control treated or treated with IFNβ for 6 days. (**E**) Real time PCR analysis of mRNAs of STAT1, STAT2, STAT3, PLSCR1, HERC6, and USP18 of MCF-7 cells, vCas9 infected wt clones, STAT2 k.o. clones, or STAT3 k.o. clones either control treated or treated with IFNβ for 4 days. Error bars represent the SD of three biological replicates. Student’s *t-*test was performed to compare gene expression between the average of four IFNβ treated parental/vCas9 controls and three STAT2 k.o. clones or three STAT3 k.o. clones. p-value *<0.05, **<0.001; ***<0.0001; ns, not significant.

We previously showed that activation of CD95 caused upregulation of IFN-I that activated a STAT1 dependent signaling pathway resulting in increased cancer stemness. This pathway could be short circuited by treating cells directly with IFN-I^29^. We also reported that in STAT1 k.o. cells neither STAT2 nor STAT3 were still phosphorylated and that addition of the JAK2 inhibitor ruxolitinib (Ruxo) to wt cells blocked activation and upregulation of STAT1 and its target genes. Consistent with an activation downstream of IFN receptors, treatment of CD95 stimulated cells with Ruxo strongly attenuated induction of the dsRNA sensors and of IRF7 (Fig. 6A). However, because STAT1 knock-out did not completely block induction of RIG-I, MDA5, TLR3, or IRF7 when stimulated with either CD95L and/or IFNβ (Fig. 6B) or of ERVs (Fig. 5B), we tested the contribution of STAT2 and STAT3 to the CD95 induced gene induction. We found that knockout of STAT2 blocked the ability of the cells to phosphorylate STAT1 (Fig. 6C), to downregulate CD24 (Fig. 6D), and to induce STAT1, STAT2 mRNAs and the main STAT1 targets PLSCR1, HERC6 and USP18 (Fig. 6E). In contrast, knockout of STAT3 barely affected the phosphorylation of either STAT1 or STAT2 (Fig. 6C). STAT3 knock-out cells showed an increased expression of STAT1, STAT2 and of the three STAT1 targets (Fig. 6E), which was further induced by the addition of IFNβ (note: the fold change was lower in STAT3 k.o. cells than in wt cells). This suggests that STAT3’s role in this pathway is antagonistic. Such antagonism is also seen in the stimulation independent upregulation of STAT3 mRNA in three STAT2 single cell k.o. clones (Fig. 6E). In contrast to STAT1 k.o. cells, STAT2 k.o. cells were completely incompetent to induce STAT1 or its target genes (Fig. 6E). Our data suggest that STAT1 and STAT2 both mediate the innate immune response seen in CD95 stimulated cells, a process that is antagonized by STAT3.

## Discussion

We recently reported that the apoptosis inducing receptor CD95 is a driver of cancer stemness under conditions of chronic stimulation^7,29^. This activity involves the release of IFN-I and activation of a STAT1 dependent gene program. Consequently, we showed in breast cancer cells that treatment with IFN-I alone also resulted in an increase in the number of stem cell markers and the ability of cells to initiate cancer *in vivo*^29^. This nonapoptotic activity of CD95 was inhibited by caspases. We now demonstrate that this novel activity of CD95 involves the canonical DISC components FADD and caspase-8 as well as activation of NF-κB. The caspase that plays the most profound role in negatively regulating this activity is caspase-2. Once inhibited, CD95 stimulation results in the production of IFN-I that after binding to IFNAR1/2 activates STAT1. STAT2 is also critical for this activation program and the activation of STAT1/STAT2 is antagonized by STAT3. Downstream in this pathway the dsRNA sensors RIG-I and MDA5 are upregulated together with a subset of ERVs. This leads to further activation of NF-κB through induction of IRF7 and phosphorylation of IRF3. While IRF3 is required for activation of NF-κB, IRF7 is not. IRF7 is also an activator of IFN-I transcription^33^. However, we did not observe an effect of knocking down IRF7 on the activation of STAT1. In contrast IRF3 knockdown attenuated CD95 induced activation of NF-κB, STAT1 signaling, and upregulation of STAT1 regulated genes. Likewise, MAVS also is known to independently drive signaling of proteins involved in this pathway and it is an activator of NF-κB^34^. However, activation of NF-κB is most important during CD95 proximal signaling, upstream of IFN-I production, as inhibiting NF-κB in CD95 stimulated cells greatly diminished production of IFN-I (Fig. 3E,F), activation of STAT1 (Fig. 3C,D) and acquisition of stemness (Fig. 3G and S5C). These data suggest the existence of a feed forward loop.

An apparent contradiction in our data was found regarding the effect the caspase-2 inhibitor had on the expression of IFN-I (Fig. 2E). Their induction was significantly reduced. Thus, while inhibiting of caspase-2 enhanced the activation of the STAT1 pathway, it resulted in reduced transcriptional activation of the three IFN-I genes. This could be explained by assuming that caspase-8 is required for the induction of IFNs and caspase-2 has two activities: it contributes to the expression of IFNs but then has an inhibitory effect on the activation of STAT1 regulated genes. It has been shown before that the pathogen sensing machinery can be inhibited by caspases and that the inhibition of caspases during lytic reactivation of Kaposi’s sarcoma-associated herpes virus strongly induced a IFN-I response^35^. The nature of the caspase involved was not entirely clear and neither was the substrate that was cleaved. However, the data suggested that caspases in part act by suppressing IFN-I induction in the canonical pathogen-sensing pathway.

It was previously shown that low dose exposure of colon cancer cells to DNA demethylating agents causes induction of dsRNA derived from ERVs^17^. These ERVs then activate the MDA5/MAVS RNA recognition pathway and IRF7. This in turn targets colorectal CICs. Interestingly, while induction of IFN-I was not observed in that study, type III IFNs IL-28a and IL-29 were strongly upregulated. In contrast, in MCF-7 cells chronically stimulated through CD95, we observed both induction of ERVs and of IFN-I. In this same model we previously showed that both CD95 stimulation and treatment with IFN-I increases cancer stemness, and that treatment with IFNβ directly increased the frequency of breast CICs *in vivo*^29^. Because we also demonstrated similar effects of CD95 stimulation and IFN-I treatment in a squamous cell carcinoma cell line^29^ and we and others showed that a IFN-I gene signature is linked to increased treatment resistance^29,36,37^, we conclude that the discrepancy between our data and the previous work on colon cancer is not due to differences between different cancers or cell lines but is more likely a reflection of the differences in the response by cells to DNA demethylating reagents and CD95 stimulation. In another report it was shown that inhibiting DNA methylation in ovarian cancer cells caused a strong upregulation of ERVs followed by dsRNA sensing and in turn triggering a IFN-I response and apoptosis^16^. Secreted IFNβ was critical to this signaling and, through interaction with surface receptors, IFNAR1/2 activated JAK/STAT signaling, transcription of ISGs, and resultant translation inhibition and apoptosis^11,38^.

In contrast to our findings, the activation of the dsRNA sensor pathway is mostly viewed as having anti-proliferative and cell death inducing activities. Such contradicting data on the activation of the IFN-I/STAT1 pathway are reminiscent to the activities of CD95. We recently implicated STAT1 to be essential for IFN-I mediated pro-tumorigenic activities of CD95^29^ and now demonstrate that STAT2 but not STAT3 is also essential for the activation the IFN-I pathway downstream of CD95. STAT2 could be part of a feed forward loop downstream of CD95 signaling. Unphosphorylated STAT2 is part of a preactivation complex with IRF9 cooperating with NF-κB and unphosphorylated ISGF3 in general is believed to prime cells for a rapid response to microbial infections^39,40^. This priming may explain the rapid increase in the response two days after stimulation of CD95. We found STAT1/2 to be part of a protumorigenic pathway while in the context of antiviral immunity they have been shown to be have antiproliferative activities^41^. However, recently, it was reported that STAT2 is required for both skin and colon carcinogenesis^42^. This is consistent with STAT2 being activated downstream of CD95 signaling, as our previous data also suggested that tumor cells cannot grow without CD95 expression^43,44^. Similar to CD95L, IFN-I have been shown multiple times to induce apoptosis in cancer cells by activating both the extrinsic and the intrinsic apoptosis pathways^45^. Data on the pro-death activities of IFNs and CD95 were initially obtained by using apoptosis sensitive cells stimulated for a short period of time with either IFN or death ligands. In contrast, in our current and recent work^7,29^ we are treating cells for 4–6 days to mimic the proinflammatory conditions cancer cells will likely encounter in a tumor where they are being exposed to both CD95L and IFN-I for long periods of time. CICs may actually not be resistant to IFN-I as previously postulated^46^ but benefit from this stimulus just like they do when exposed to CD95L long term.

## Material and Methods

### Cell line and reagents

The MCF-7, Hs578T and HCC70 were purchased from the ATCC and these cells were cultured in RPMI 1640 medium (Mediatech Inc) containing 10% heat-inactivated fetal bovine serum (FBS) (Sigma-Aldrich) and 1% penicillin/streptomycin (Mediatech Inc). SCC-61 were described previously^29^. They were grown in DMEM/F-12 (1:1) (Gibco) with 20% FBS and 1% penicillin–streptomycin, 0.4 μg/ml hydrocortisone (Sigma Aldrich). MCF-7-Fas-vector and MCF-7-Fas-Bcl-x_L_ were cultured as previously described^47^. The source and dilution of primary antibodies of anti-STAT1, anti-pY701STAT1, anti-STAT2, anti-pY690STAT2, anti-STAT3, anti-pY705STAT3, anti-PLSCR1 and HRP conjugated anti ß-actin antibody were described previously^29^. Antibodies to human CD95 (sc-715, C-20) and anti-HA (sc-7392) were from Santa Cruz, anti-RIP1 (#610458) and anti-FADD (#610400) were from BD Bioscience. Anti-pIκBα (#9246), anti-IκBα (#4814), anti-Bcl-x_L_ (#2764), anti-RIG-I (#4200), anti-MDA5 (#5321), anti-MAVS (#3993), anti-pTBK1 (#5483), anti-TBK1 (#3504), anti-TLR3 (#6961), anti-IRF3 (#11904), anti-pIRF3 (#4947), anti-IRF7 (#4920), anti-pIRF7 (#5184), anti-caspase-2 (#2224) were from Cell Signaling. The anti-cGAS (#ABF124) antibody was from Millipore. The anti-caspase-8 (C-15) antibody was previously described^48^. Anti-human CD95 and anti-caspase-8 were used at 1:500, all other primary antibodies at 1:1000, unless otherwise specified. All secondary antibodies were used at 1:5000: goat anti-mouse IgG, HRP-conjugated was from Cell Signaling, rabbit anti-goat IgG, HRP-conjugated, and goat anti-rabbit IgG, HRP-conjugated was from Southern Biotech.

The NF-κB inhibitor BAY 11–7082 (B5556), bovine serum albumin, hydrocortisone, puromycin, DMSO and the RIP1 inhibitor necrostatin1 were purchased from Sigma Aldrich. Caspase-2 inhibitor zVDVAD (FMK003), caspase-3/7 inhibitor zDEVD (FMK004), caspase-6 inhibitor zVEID (FMK006), caspase-8 inhibitor zIETD (FMK007), caspase-9 inhibitor zLEHD (FMK008) and caspase-10 inhibitor zAEVD (FMK009) were from R&D Systems. zVAD-fmk (BML-P416) was from Enzo Lifesciences. All caspase inhibitors were used at 20 μM concentration. Ruxolitinib (sc-36472) (used at 20 μM) was from Santa Cruz Biotechnology. Blasticidin was purchased from InvivoGen.

Anti-CD95 mAb anti-APO-1, leucine zipper tagged CD95L (LzCD95L) were used at 100 ng/ml as described before unless otherwise specified^29^. In all cases when anti-APO-1 or control IgG3 was used 1 ng/ml of Protein A (Sigma) was added to help crosslink the antibodies. IFNβ (11415–1) (used at 1000 U/ml) were purchased from pbl Assay Science. Cells treated with inhibitors were pretreated at least 1 hr before for any further treatment of anti-APO-1, LzCD95L, or IFNβ.

### FACS and Western blot analysis

Flow cytometry and Western blot analysis were performed as previously described^29^.

### Sphere forming assay

Cells were pre-treated with IgG3, anti-APO-1, LzCD95L, IFNβ, or received no treatment for 6 days. Cell suspensions were passed through a 40 μm sterile cell strainer (Fisher Scientific) to obtain single cell suspension. The strained cell suspension was diluted in Mammocult media and seeded at 5000 cell/well in triplicate in ultra-low adherence 24-well plates (Corning). The cells were cultured in Mammocult media (Cell Stem Technology; prepared according to manufacturer’s instructions) supplemented with 4 μg/ml Heparin, 0.5 μg/ml hydrocortisone and 10% Mammocult Proliferation Supplement. Spheres were counted using a light microscope 6 days after seeding.

### ELISA

3 × 10^5^ MCF-7 cells were seeded in 6 well plates and then cells were pretreated for 1 hr with DMSO or 5 μM of BAY11-7082 before adding LzCD95L for 48 hr. Cells and cell supernatant were harvested and combined. ELISA was performed as described previously^29^.

### Quantitative real-time PCR

Total RNA extraction, RNA quantification and cDNA preparation were done as previously described^29^. Primers used to quantify GAPDH, STAT1, PLSCR1, HERC6 and USP18 were previously described^29^. The expression of STAT2 (Hs01013115_g1), STAT3 (Hs00374280_m1), IFNB1 (Hs01077958_s1), IFNA1 (Hs04189288_g1), IFNA2 (Hs00265051_s1), ISG15 (Hs01921425_s1), RIG-I (Hs01061436_m1), MDA5 (Hs00223420_m1), TLR3 (Hs01551079_g1), MAVS (Hs00920075_m1), cGAS (Hs00403553_m1), TBK1 (Hs00179410_m1), STING (Hs00736955_g1), IRF3 (Hs01547283_m1) and IRF7 (Hs01014809_g1) was quantified using specific primers from Life Technologies.

Real-time PCR for ERV genes was performed using the Applied Biosystems PowerUp SYBR Green Master Mix. The forward and reverse primers for the amplification of ERV were described previously^17^. The primer sequence were used as follows (5′ → 3′): MER21C, GGAGCTTCCTGATTGGCAGA and ATGTAGGGTGGCAAGCACTG; MLT1C49, TATTGCCGTACTGTGGGCTG and TGGAACAGAGCCCTTCCTTG; ERVL, ATATCCTGCCTGGATGGGGT and GAGCTTCTTAGTCCTCCTGTGT; MER4D, CCCTAAAGAGGCAGGACACC and TCAAGCAATCGTCAACCAGA; GAPDH, CCTCAACGACCACTTTGTCA and CCCTGTTGCTGTAGCCAAAT. The expression levels of target genes were normalized to the reference housekeeping gene, GAPDH. Fold differences were then calculated for each treatment group using normalized *C*_*T*_ values for the control. The qPCR was run either in 96 well qPCR plates with 20 μl of reaction mixtures in AB 7500 Fast Real-Time system or in 384 well plates with 10 μl reaction mixtures in QuantStudio Real-Time PCR System from Applied Biosystems.

### Gene silencing using lentiviral shRNAs or siRNAs

To knockdown IRF3 and IRF7 using siRNAs, 2 × 10^5^ MCF-7 cells/well were seeded in 6 well plates one day prior to transfection. Cells were transfected with either 50 nM of a SMART pool of 4 mammalian nontargeting siRNAs (D-001810-10-20) (UGGUUUACAUGUCGACUAA, UGGUUUACAUGUUGUGUGA, UGGUUUACAUGUUUUCUGA, UGGUUUACAUGUUUUCCUA) or 50 nM of ON-TARGETplus Human IRF3 siRNA - SMARTpool (L-006875-00-0005) or ON-TARGETplus Human IRF7 siRNA - SMARTpool IRF7 (L-011810-00-0005) using Dharmafect 2. After 24 hrs, the transfected cells were either treated with LzCD95L or IFNβ or left untreated for 4 days.

To knockdown MAVS,150,000 MCF-7 cells seeded in 6 well plates were infected with MISSION Lentiviral Transduction Particles (Sigma): either pLKO.2-puro control transduction particle coding for a nontargeting (scrambled) shRNA (#SHC002) (CCGGCAACAAGATGAAGAGCACCAACTCGAGTTG GTGCTCTTCATCTTGTTGTTTTT) or shRNAs against mRNA *Homo sapiens* MAVS TRCN0000236031 (CCGGATGTGGATGTTGTAGAGATTCCTCGAGGAATCTCTACAACATCCACATTTTTTG) at a M.O.I. (Multiplicity of infection) of 3 in the presence of 8 μg/ml polybrene. Cells were selected with puromycin as previously described^44^.

### Deletion of CD95, FADD, caspase-8, caspase-2, STAT1, STAT2 and STAT3 gene

The generation of STAT1 k.o. in MCF-7 (S1-6 and S1-10, formerly described as clone P6 and P10, respectively) was described previously^29^. The complete CD95 deletion (clone F1) and CD95 exon 4 specific deletion in MCF-7 cells (clone F2, formerly described as #R6-3) was also described previously^49^. To generate FADD and caspase-8 k.o. MCF-7 cells two gRNAs were designed for both FADD (gRNA#1: TTCCTATGCCTCGGGCGCGTGGG and gRNA#2: GGGAGG CGAGCAGCTCGCGCAGG) and caspase-8 (gRNA#1: AACATCAAGGCATCCTTGATGGG and gRNA#2: TACCTACCTAAACACTAGAAAGG) to delete a section covering part of exon1 in the FADD gene and part of exon2 in the caspase-8 gene, respectively. The entire DNA fragments were synthesized as gBlocks (U6 promoter + target sequence + guide RNA scaffold + termination signal) by IDT. gBlock pairs were transfected together with a Cas9 plasmid (pMJ920 plasmid coding for GFP) into 2 × 10^5^ MCF-7 cells using Lipofectamine. Two days after transfection, 10–20% of cells that showed the highest GFP expression were sorted by FACS and expanded in 6 well plates. After one week cells were subjected to cell sorting at 1 cell per well directly into 96-well plates. After approximately two to three weeks, single cell clones were expanded and subjected to genotyping. To identify complete genomic deletions caused by gRNA pairs, genomic PCR was performed with a pair of primers (forward: 5-GCGAGATAACGGTCGAAAAC-3 and reverse: 5-ATATTCCGAGACAGAAAGGAAATG-3) for the FADD and forward: 5-CAGCAAATGGTACTTTTCTTCCTT-3 and reverse: 5-ACTAGGCAGGGTACCACAAAAA-3 for the caspase-8. Internal primer pairs (forward: 5-CAGCAAATGGTACTTTTCTTCCTT-3 and reverse: 5-TCTCTTTTCCTGGAGTCTCTGG-3) were used to screen for single cell clones of caspase-8 k.o that harbor a homozygous deletion. Complete absence of FADD or caspase-8 protein in different FADD and caspase-8 k.o. clones was confirmed by Western blot.

To generate MCF-7 caspase-2, STAT2 and STAT3 k.o. cells, caspase-2, STAT2 and STAT3 specific gRNA sequences were designed to efficiently target with minimal risk of off-target Cas9 binding elsewhere in the genome, as described elsewhere^50^. We first established lentiCas9-Blast-MCF-7 cells (vCas9-MCF-7) lines with stable expression. The lentiCas9-Blast virus was obtained from the viral core facility at Northwestern University. MCF-7 cells (10^5^/well) were plated in 6 well plates and on the next day cells were infected with the indicated lentiCas9-Blast virus supernatants with polybrene (final concentration: 8 μg/ml). The culture supernatants were replaced with fresh medium containing antibiotics 48 hours after transduction. Uninfected cells were eliminated by culturing in selection medium RPM1-1640 + selection marker Blasticidin at an end-concentration 5 μg/ml.

Lentiviral vectors expressing single guide RNAs (sgRNAs) targeting caspase-2, STAT2 and STAT3 were generated according to the lentiGuide-Puro (two vector system) protocol^50^. LentiGuide-Puro plasmid (Addgene #52963) was a gift from Feng Zhang^50^. The guide RNA without PAM sequence used were: ATCGTGGCACTCCTCTCGCA (vCasp2-gRNA1) and CGATGCAGGAGTCCGTGACT (vCasp2 gRNA2) for caspase-2, TGGCGGATGATTTCAGTCAG (vSTAT2-gRNA) for STAT2, GAAACTGCCGCAGCTCCATT (vSTAT3-gRNA) for STAT3. The gRNA sequences were synthesized and inserted into LentiGuide-Puro vector as described^50^. After insertion of the specific gRNA into the plasmid, it was transformed into competent *E. coli* (Stbl3, Invitrogen). Plasmids were isolated from a single colony. The lentiviral vectors were co-transfected with packaging vectors psPAX2 (Addgene #12260) and pCMV-VSV-G (Addgene #8454) into 293 T cells for lentivirus production. To obtain a k.o. pool, vCas9-MCF-7 cells were transduced using the above lentiviruses with polybrene (8 μg/ml). Forty-eight hours after infection, cells were selected with 3 μg/ml puromycin for 3–5 days. Caspase-2 k.o. pool generated by vCasp2-gRNA2 was used for further experiments after evaluation of decreased in protein expression of caspase-2 by Western blot. Once evaluated for overall editing efficiency by Western blot, STAT2 and STAT3 k.o. pool generated by the vSTAT2-gRNA and vSTAT3-gRNA, respectively were used to isolate single clones in 96 well plates to get complete k.o. clones.

### Metascape analysis

The functional and pathway enrichment analysis was performed using the Metascape tool (www.metascape.org) with all genes that were up or downregulated at least 1.0 (log2) fold in MCF-7 cells stimulated through CD95 for 2 weeks as previously reported^29^. Gene array files can be downloaded from the Gene Expression Omnibus, GEO: GSE81860. For each given gene list, pathway and process enrichment analysis was carried out with the Reactome Gene Set. All genes in the genome were used as the enrichment background. Terms with a p-value < 0.01, a minimum count of 3, and an enrichment factor > 1.5 were collected and grouped into clusters based on their membership similarities.

### Overexpressing a dominant negative mutant IκBα

Transient transfections of pCMV4–3HA control (Addgene #24165) or pCMV4–3 HA/IκB-alpha (Addgene #21985) or dominant negative mutant IκBα pCMV4–3 HA/IκB-alpha (SS32,36AA) (Addgene #24143) was performed using Lipofectamin 2000 (Invitrogen). After an overnight incubation, the culture medium was replaced with fresh medium with or without 20 μM zVAD. After 1 hr of zVAD treatment LzCD95L was added to treated samples.

### Reconstitution of caspase-8 k.o. clones with mutant caspase-8

A lentiviral vector expressing human catalytically inactive-caspase-8 (caspase-8 (C360A)-pcw107-V5) was a gift from David Sabatini & Kris Wood (Addgene plasmid # 64632)^51^. To generate viruses that express mutant caspase-8 (C360A)-pcw107-V5, 3 μg pCMVDR8.9 (packaging), 3 μg pMD2.G (envelope), and 6 μg of the lentiviral vector expressing human caspase-8 (C360A)-pcw107-V5 were transfected in 293 T cells using Lipofectamine 2000 (Invitrogen). Viruses were generated as previously described^29^. The supernatant medium containing pLenti-human mutant caspase-8 was collected after 48 h and, after filtering through a 0.22 µm syringe filter (Millipore), was used to infect two caspase-8 k.o. clones (C8–11 & C8–17) with 8 μg/ml polybrene. Selection was done with 3 μg/mL puromycin for more than 2 weeks.

### Statistical methods

The results from qPCR, sphere formation assay and ELISA were presented as the mean ± SD and analyzed by the Student’s two-tailed t test. Statistical significance was defined as p < 0.05. A linear model for continuous gene expression levels, using binary predictors (cell stimulus and an inhibitor their interaction term) was used to evaluate whether the effect of a treatment gene expression varied depending on the presence of an inhibitor using STATA14. Statistical significance of the interaction term at p < 0.05 was used to identify this difference.

## Supplementary information

Supplementary Information.

## Data Availability

All data needed to evaluate the conclusions in the paper are present in the paper or the Supplementary Materials.
