# Supplementary material for: The mechanism of how CD95/Fas activates the Type I IFN/STAT1 axis, driving cancer stemness in breast cancer
Source: Sci Rep. 2020 Jan 28;10:1310. doi: 10.1038/s41598-020-58211-3 (PMC6987111; doi:10.1038/s41598-020-58211-3)
Supplement: Supplementary file 1 — Supplementary Information. [file 41598_2020_58211_MOESM1_ESM.pdf]

Supporting Information for

The mechanism of how CD95/Fas activates the Type I IFN/STAT1 axis, driving cancer stemness in breast cancer

Abdul S. Qadir[1,3], Austin M. Stults[1], Andrea E. Murmann[1], Marcus E. Peter[1,2]*

[1] Division Hematology/Oncology and [2] Department of Biochemistry and Molecular Genetics, Feinberg School of Medicine, Northwestern University, Chicago, IL 60611, USA

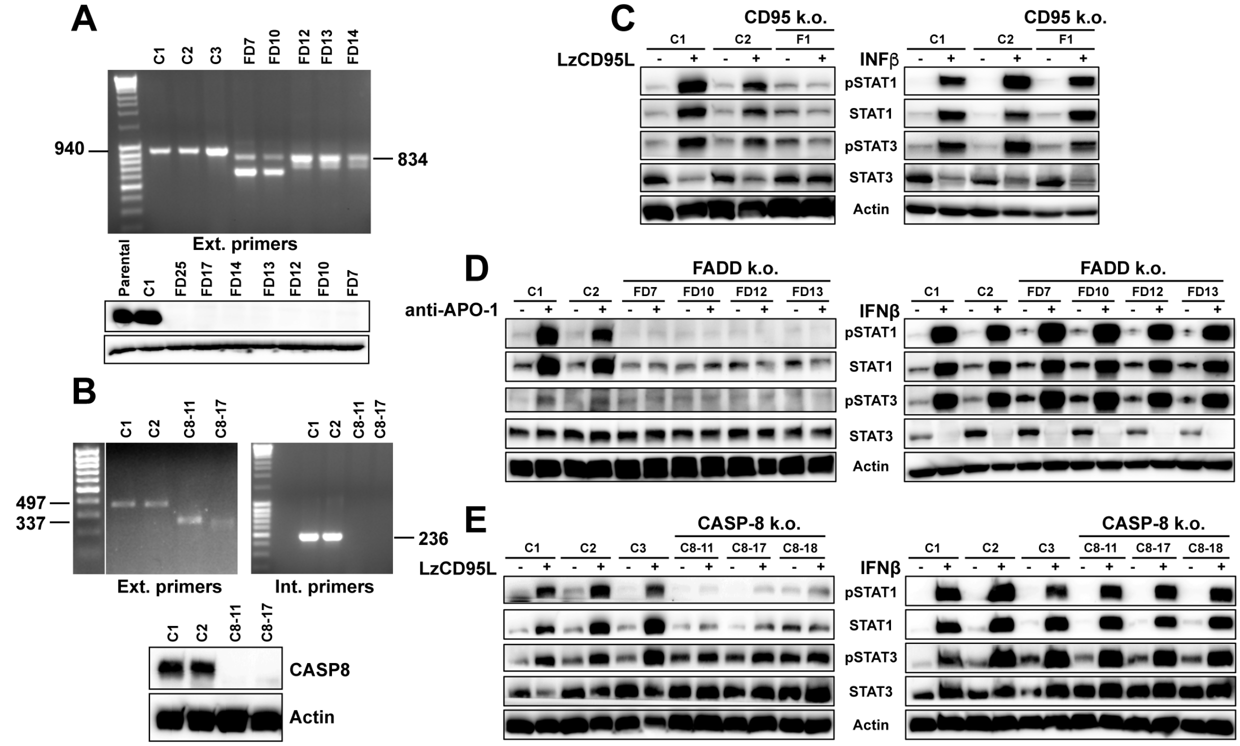

Figure S1. Deletion of CD95, FADD, or caspase-8 blocks STAT1 phosphorylation during prolonged stimulation of CD95. (**A**) PCR confirmation of homozygous deletions (Δ106 bp) in FADD in five isolated clones (upper panel) and western blot analysis confirming absence of FADD protein in different k.o. clones. Clones transfected with Cas9 only (C1, C2 and C3) were used as a wt control for PCR and MCF-7 parental and Cas9 clone (C1) were used as a wt controls for the western blot analysis. (**B**) PCR with external primers (upper left panels) and internal primers (upper right panels) used to confirm caspase-8 k.o. in two isolated clones (C8-11 and C8-17) and western blot analysis (lower panel) confirming absence of caspase-8 protein in both caspase-8 k.o. clones. Clones transfected with Cas9 only (C1 and C2) were used as wt. (**C**) Western blot analysis of Cas9 transfected wt clones (C1 and C2) and a complete CD95 k.o. clone (F1) of MCF-7 either control treated or treated with LzCD95L (left panel), or with IFNβ (right panel) for 4 days. (**D**) Western blot analysis of Cas9 transfected wt clones (C1 and C2), FADD k.o. clones (FD7, FD10, FD12, and FD13) of MCF-7 either control treated or treated with anti-APO-1 (left panel) or IFNβ (right panel) for 4 days. (**E**) Western blot analysis of Cas9 transfected wt clones (C1, C2 and C3) and caspase-8 k.o. clones (C8-11, C8-17 and C8-18) of MCF-7 either control treated or treated with LzCD95L (left panel) or IFNβ (right panel) for 4 days. All uncropped immunoblot images are included in Fig. S9.

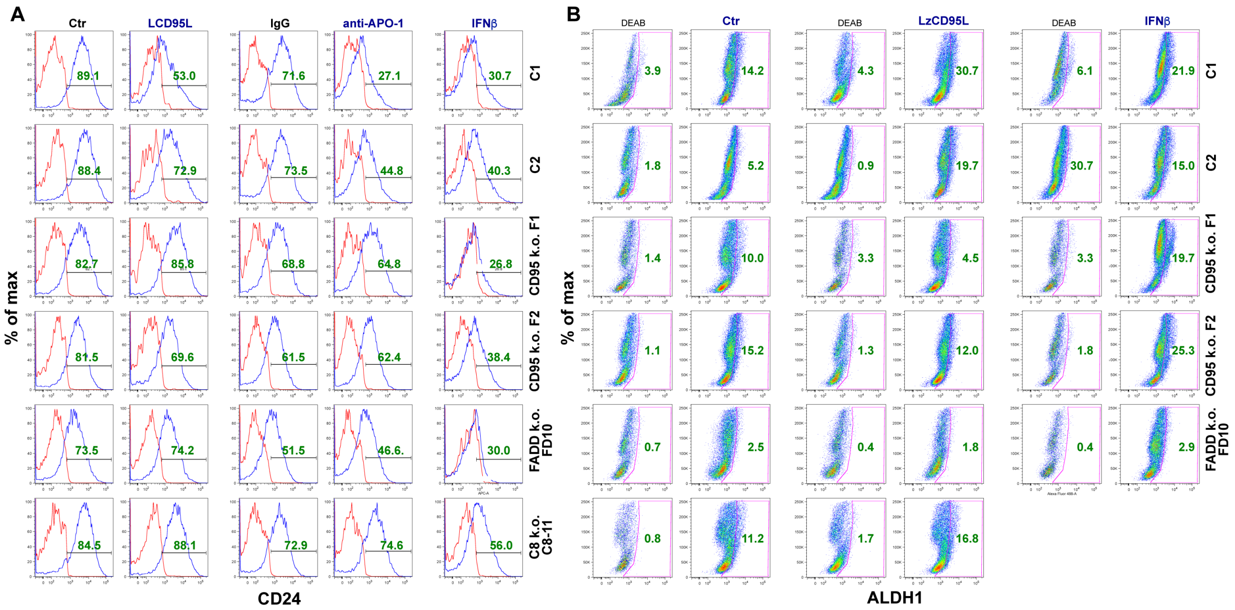

Figure S2. Knock-out of CD95, FADD, or caspase-8 in MCF-7 cells blocks CD95 stimulation mediated cancer stem cell formation. (**A,B**) CD24 surface staining (A) and ALDH1 assay (B) of Cas9 transfected wt clones (C1 and C2) and CD95 (F1-complete CD95 k.o., F2-CD95 exon 4 deletion), FADD (FD10) and caspase-8 (C8-11) k.o. clones of MCF-7 either untreated control (Ctr) or treated with LzCD95L or IgG3 or anti-APO-1 or IFNβ for 6 days. Data for caspase-8 k.o. (C8-11) clone in the ALDH1 assay treated with IFNβ was not available.

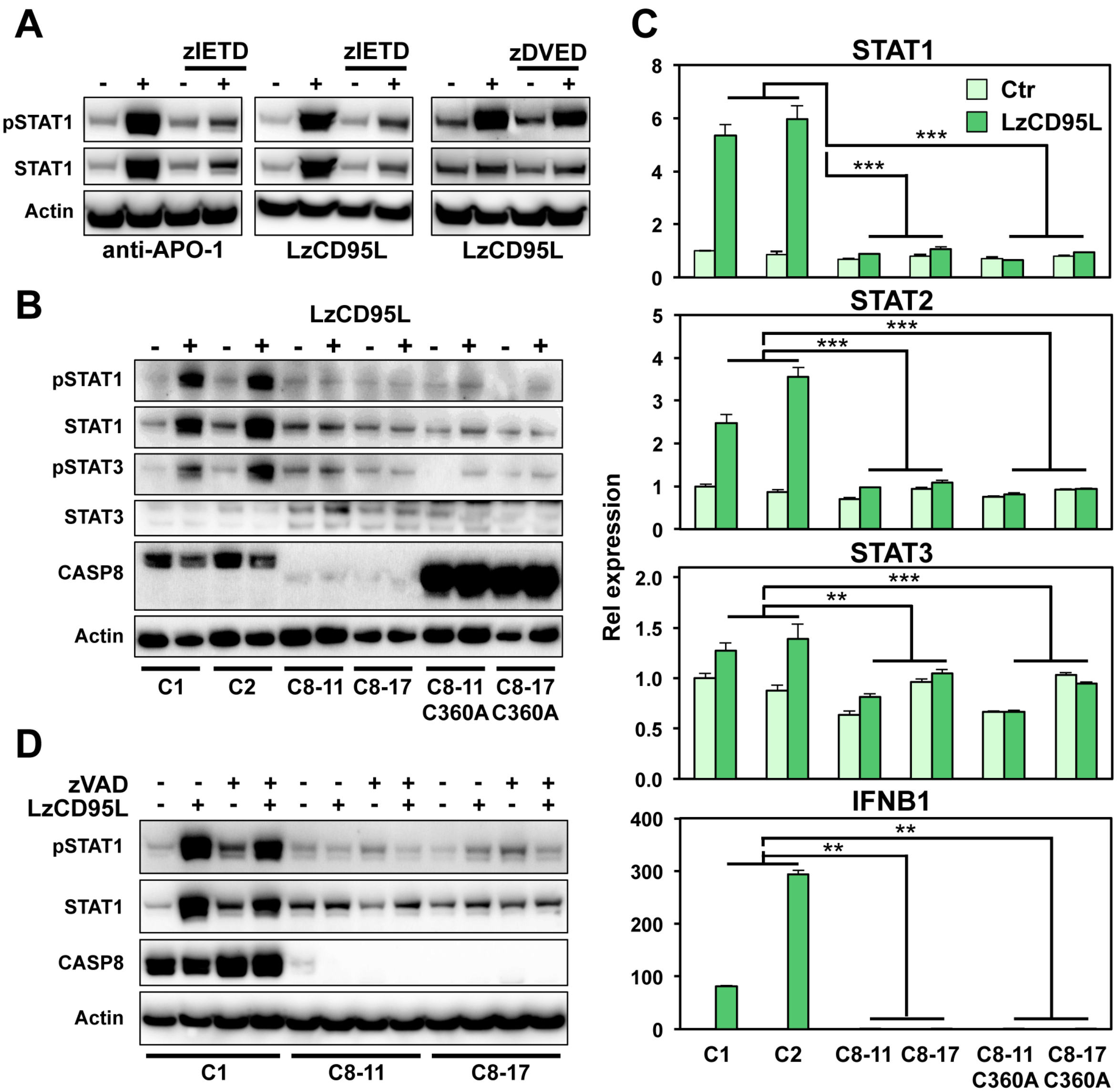

Figure S3. Dependence on caspase-8 of STAT1 activation during CD95 stimulation. (**A**) Western blot analysis of MCF-7 cells treated with solvent control or with 20 μM of the caspase-8 inhibitor (zIETD), with anti-APO-1 (left panel), or LzCD95L (middle panel) or 20 μM of the caspase-3/7 inhibitor (zDEVD) upon LzCD95L treatment (right panel) for 4 days. (**B**) Western blot analysis of Cas9 clones, two caspase-8 k.o. clones, or two caspase-8 k.o. clones reconstituted with a catalytically inactive caspase-8 gene (C8-11-C360A and C8-17-C360A) treated with LzCD95L for 4 days. (**C**) Real-time PCR quantification of mRNAs of STAT1, STAT2, STAT3 and IFNB1 of the clones treated as in B. Error bars represent the SD of three biological replicates. Student's *t*-test was performed to compare gene expression between the average of two LzCD95L treated controls with the average of two caspase-8 k.o. clones or the average of the k.o. clones reconstituted with catalytically inactive caspase-8. p-value **<0.001; ***<0.0001. (**D**) Western blot analysis of a Cas9 clone and two caspase-8 k.o. clones in the presence of solvent control or 20 μM of the oligo caspase inhibitor (zVAD) upon treatment with LzCD95L for 4 days. All uncropped immunoblot images are included in Fig. S9.

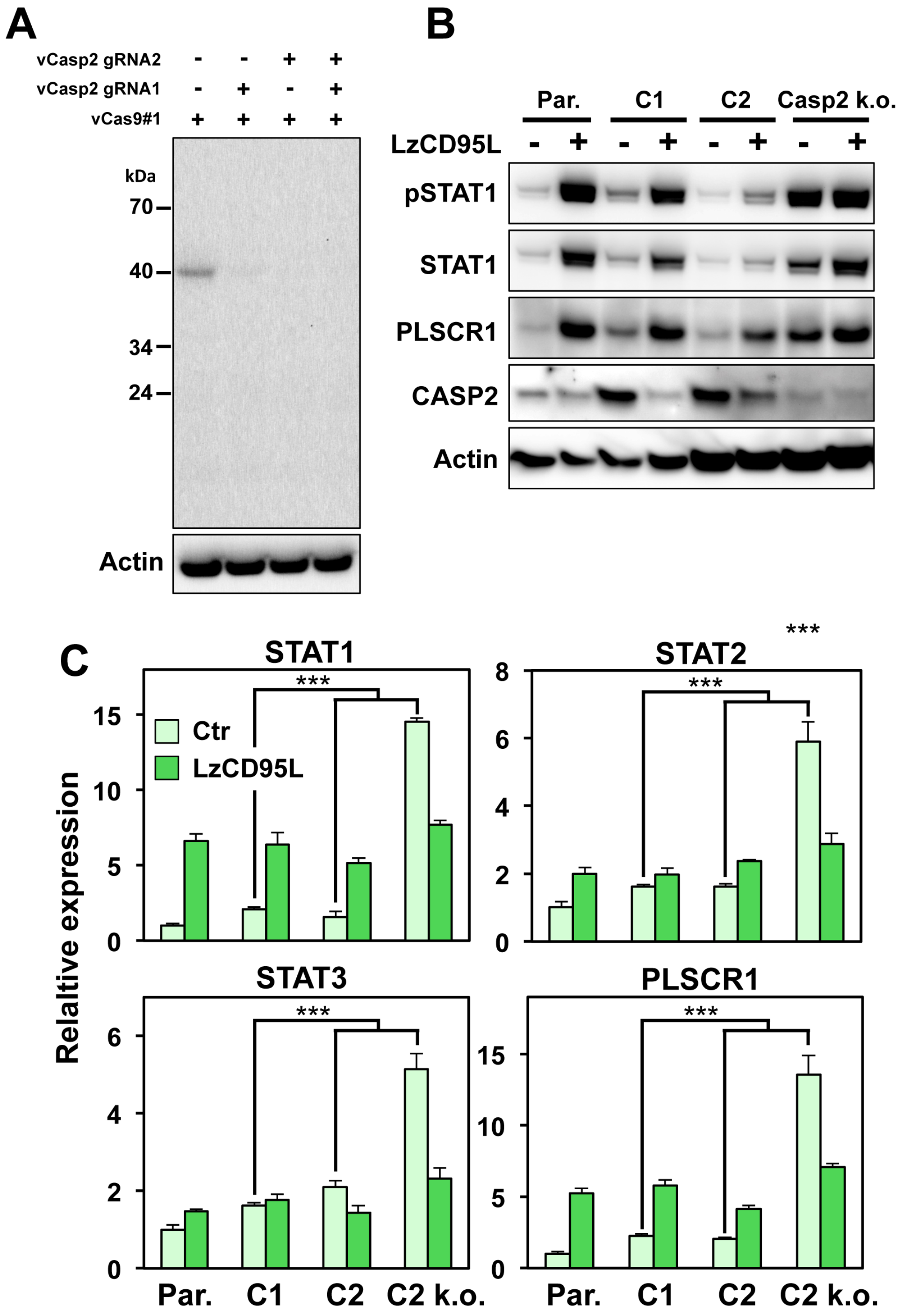

Figure S4. Deletion of caspase-2 using CRISPRi induces STAT1 activation. (**A**) Western blot analysis of MCF-7 cells either infected with a vCas9 vector (vCas9#1) or infected with vCas9 and different gRNA vectors targeting caspase-2. (**B**) Western blot analysis of MCF-7 parental, two Cas9 clones (C1 and C2) or a pool of caspase-2 k.o. cells infected with viral caspase-2 gRNA2 upon LzCD95L treatment for 4 days. All uncropped immunoblot images are included in Fig. S9. (**C**) Real-time PCR quantification of mRNAs of STAT1, STAT2, STAT3, and PLSCR1 of the cells treated as in B. Error bars represent the SD of three biological replicates. Student's *t*-test was performed to compare gene expression between the average of two unstimulated controls and unstimulated caspase-2 k.o. pool. p-value ***<0.0001.

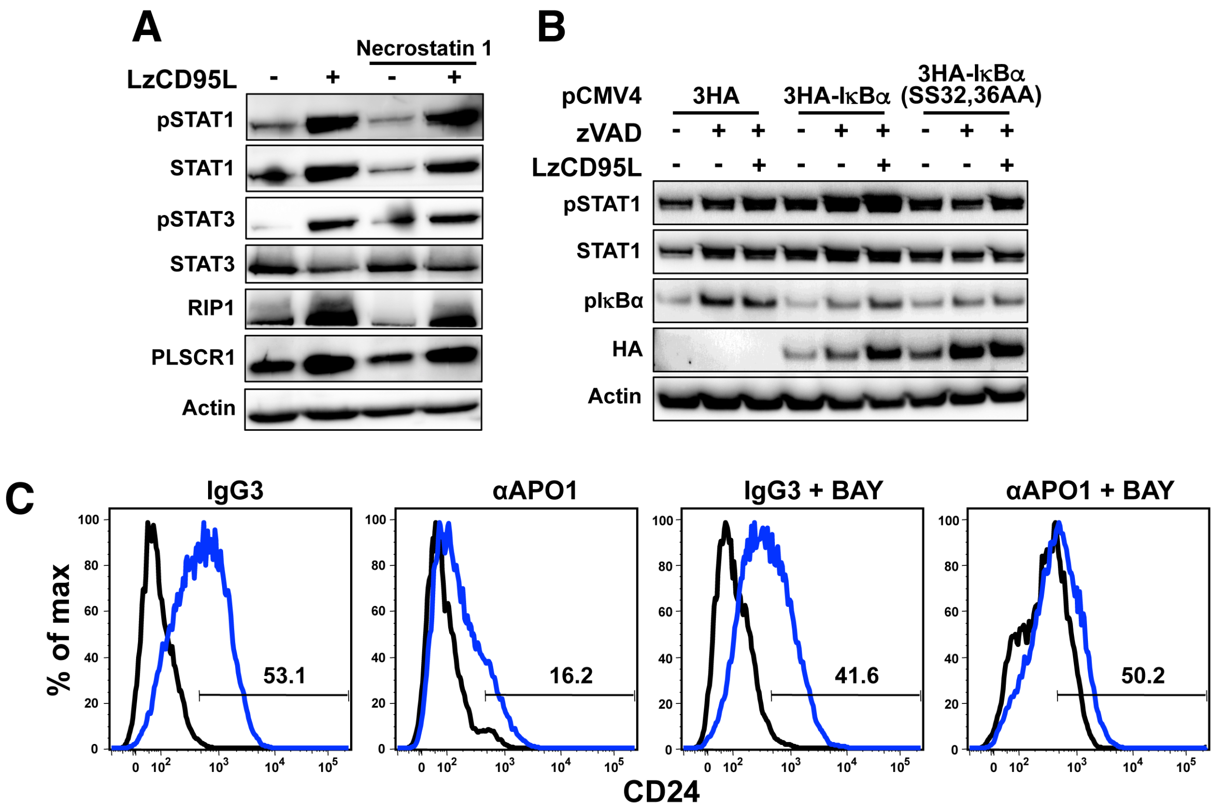

Figure S5. NF-κB activation but not RIP1 signaling is required for CD95 mediated STAT1 activation and CSC formation. (**A**) Western blot analysis of MCF-7 cells treated with solvent control or with 20 μM of Necrostatin-1 control treated or treated with LzCD95L for 4 days. (**B**) Western blot analysis of MCF-7 cells transfected with control vector pCMV4-3 HA or pCMV4-3 HA/IκBα or pCMV4-3 HA/IκBα (SS32,36AA) upon treatment with zVAD exposed to LzCD95L for 4 days. All uncropped immunoblot images are included in Fig. S9. (**C**) CD24 surface staining of MCF-7 cells treated with solvent control or with 5 μM of BAY 11-7082 control treated (IgG3) or treated with anti-APO-1 (αAPO-1) for 6 days.

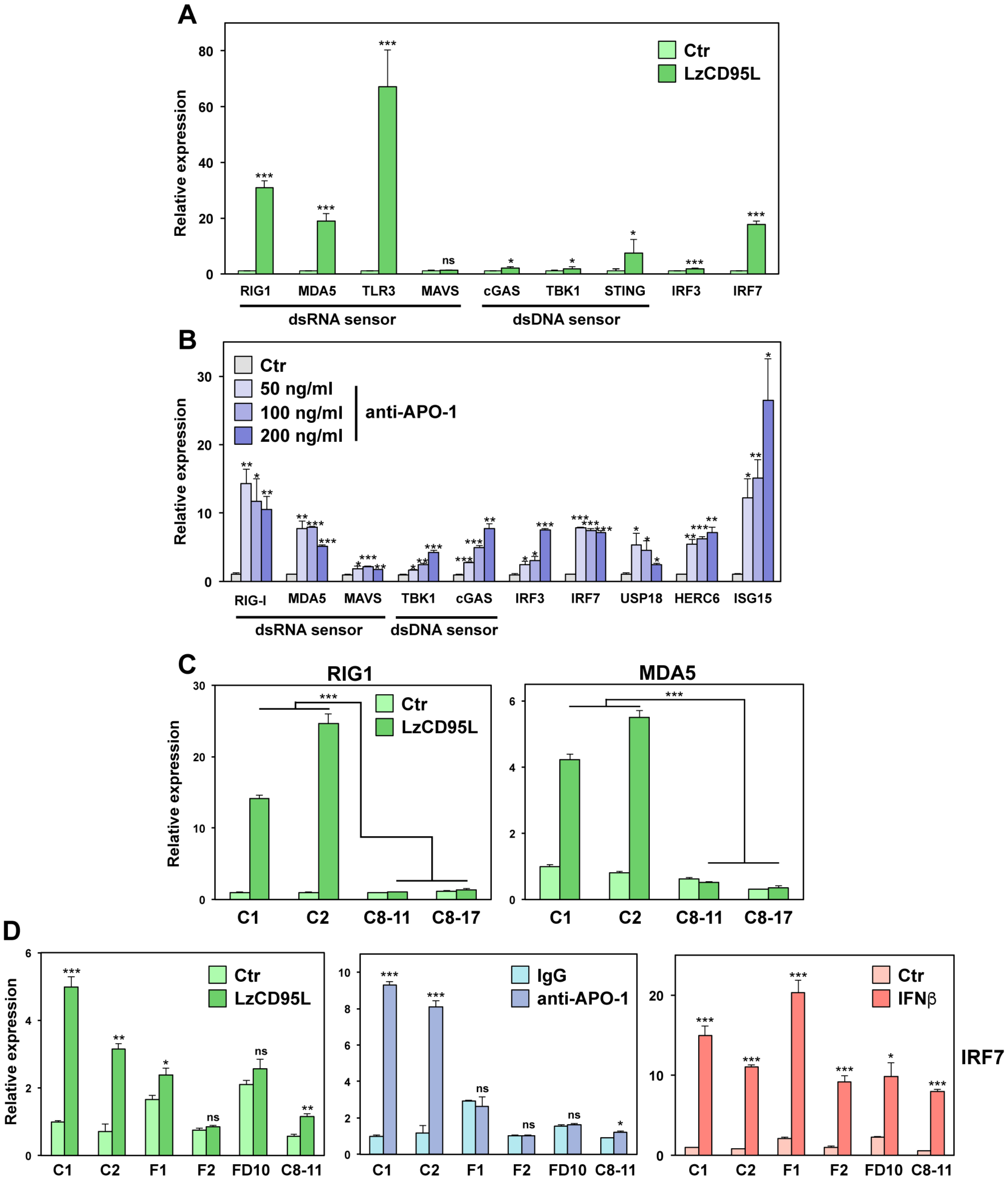
A
Relative expression
Ctr
LzCD95L
RIG1
MDA5
TLR3
MAVS
cGAS
TBK1
STING
IRF3
IRF7
dsRNA sensor
dsDNA sensor
B
Ctr
50 ng/ml
100 ng/ml
200 ng/ml
anti-APO-1
RIG-I
MDA5
MAVS
TBK1
cGAS
IRF3
IRF7
USP18
HERC6
ISG15
dsRNA sensor
dsDNA sensor
C
RIG1
MDA5
C1
C2
C8-11
C8-17
D
IgG
anti-APO-1
IFNβ
F1
F2
FD10
IRF7

Figure S6. Prolonged stimulation of CD95 induces expression of dsRNA sensors. (**A**) Real time PCR analysis of mRNAs of dsRNA sensors, dsDNA sensors, IRF3 and IRF7 in MCF-7 cells control treated or exposed to LzCD95L for 4 days. Error bars represent the SD of four biological replicates. Student's *t*-test was performed comparing samples with matching controls. (**B**) Real time PCR analysis of mRNAs of dsRNA sensors, dsDNA sensors, IRF3, IRF7 and STAT1 target genes USP18, HERC6 and ISG15 in MCF-7 cells IgG3 control treated (Ctr) or exposed to different concentration of anti-APO-1 for 4 days. Error bars represent the SD of three biological replicates. Student's *t*-test was performed to compare with their IgG3 treated controls (Ctr). (**C**) Real time PCR analysis of RIG-I and MDA5 of two Cas9 control clones (C1 and C2) and two Caspase-8 k.o. clones 4 days after treatment with either LzCD95L or left untreated (Ctr). Error bars represent the SD of three biological replicates. Student's *t*-test was performed to compare gene expression between average of two LzCD95L treated controls and two caspase-8 k.o. clones. (**D**) Real-time PCR quantification of mRNAs of IRF7 of Cas9 control clones (C1 and C2), CD95 (F1-complete CD95 k.o., F2-CD95 exon 4 deletion), FADD (FD10), or caspase-8 (C8-11) k.o. clones of MCF-7 treated with either LzCD95L (left panel), anti-APO-1 (middle panel), or IFNβ (right panel) for 4 days. Error bars represent the SD of three biological replicates. Student's *t*-test was performed compare with matching controls. p-value *<0.05, **<0.001; ***<0.0001; ns, not significant.

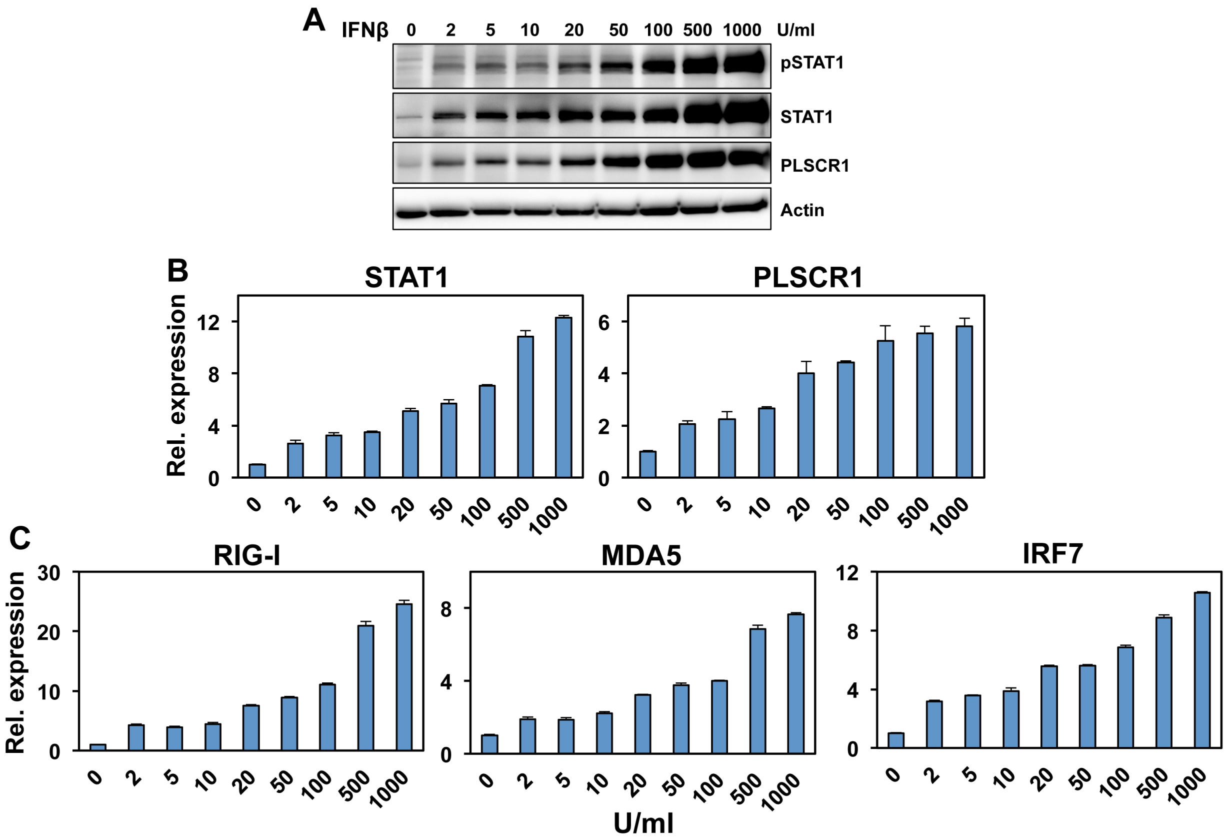

Figure S7. Dose dependence of dsRNA sensors and STAT1 activation in IFNβ stimulated cells. (**A**) Western blot analysis of MCF-7 cells treated with different concentration of IFNβ for 4 days. All uncropped immunoblot images are included in Fig. S9. (**B, C**) Real time PCR analysis of mRNAs of STAT1, and PLSCR1, RIG-I, MDA5, and IRF7 in MCF-7 cells treated with different concentration of IFNβ for 4 days. Error bars represent the SD of four biological replicates.

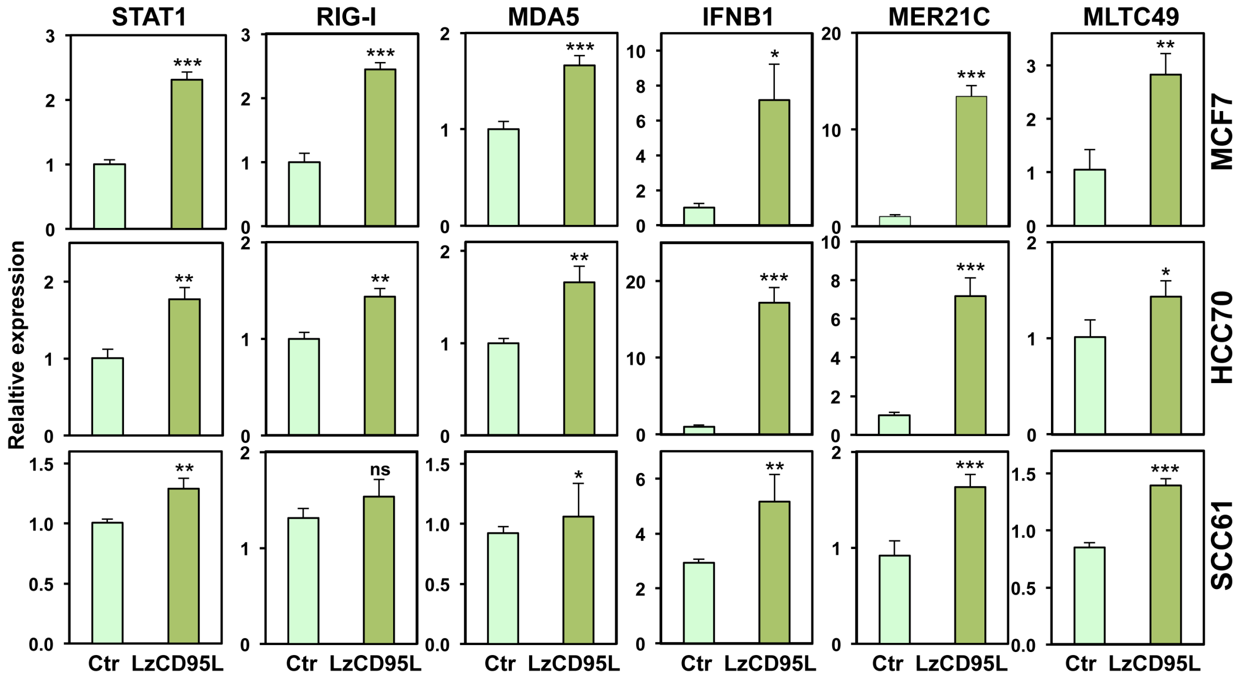

Figure S8. dsRNA sensor and ERV expression in MCF-7, HCC70 and SCC61 cells upon CD95 stimulation. Real time PCR analysis of mRNAs of STAT1, RIG-I, MDA5, IFNB1, MER21C, and MLTC49 in MCF-7, HCC70 cells treated with LzCD95L without zVAD or SCC61 cells treated with LzCD95L in the presence of 20 μM zVAD for 4 days. Error bars represent the SD of four biological replicates. Student's *t*-test was performed comparing samples to matching controls. p-value *<0.05, **<0.001; ***<0.0001; ns, not significant.

Figure 1

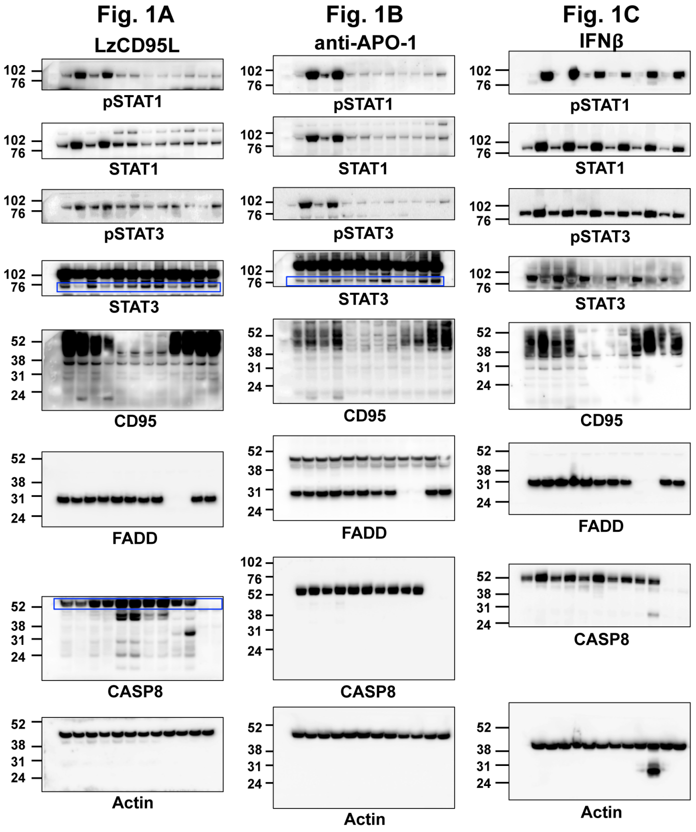

Figure 2

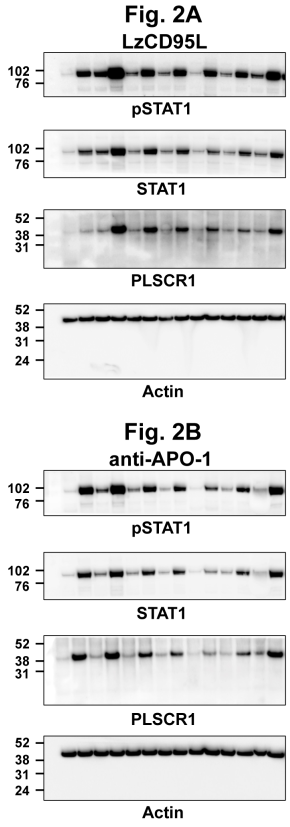

Figure 3

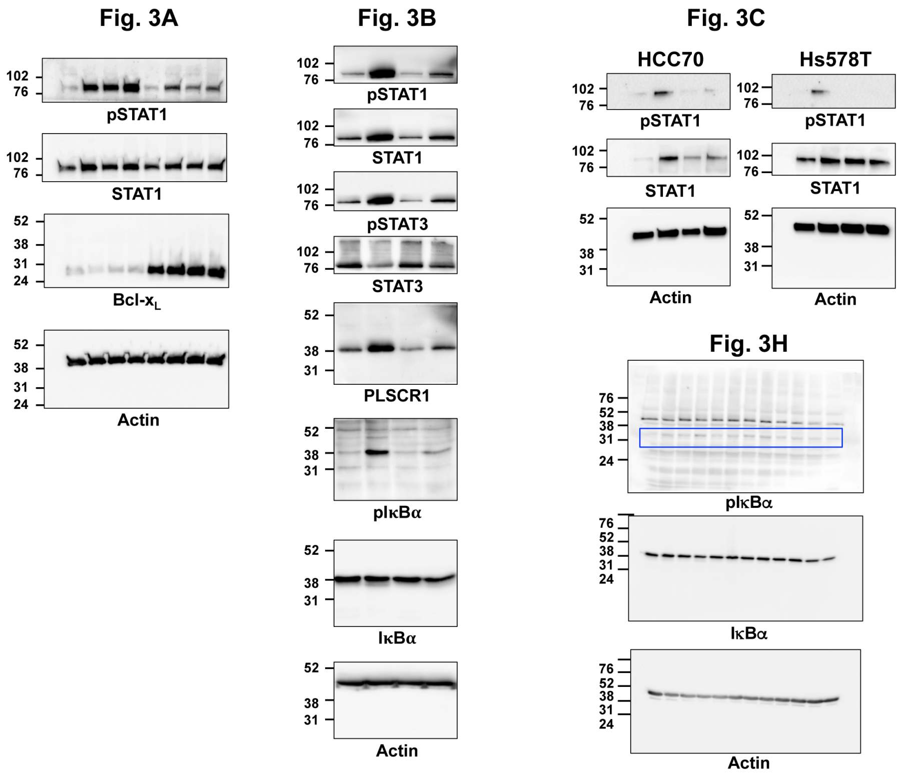

Figure 4

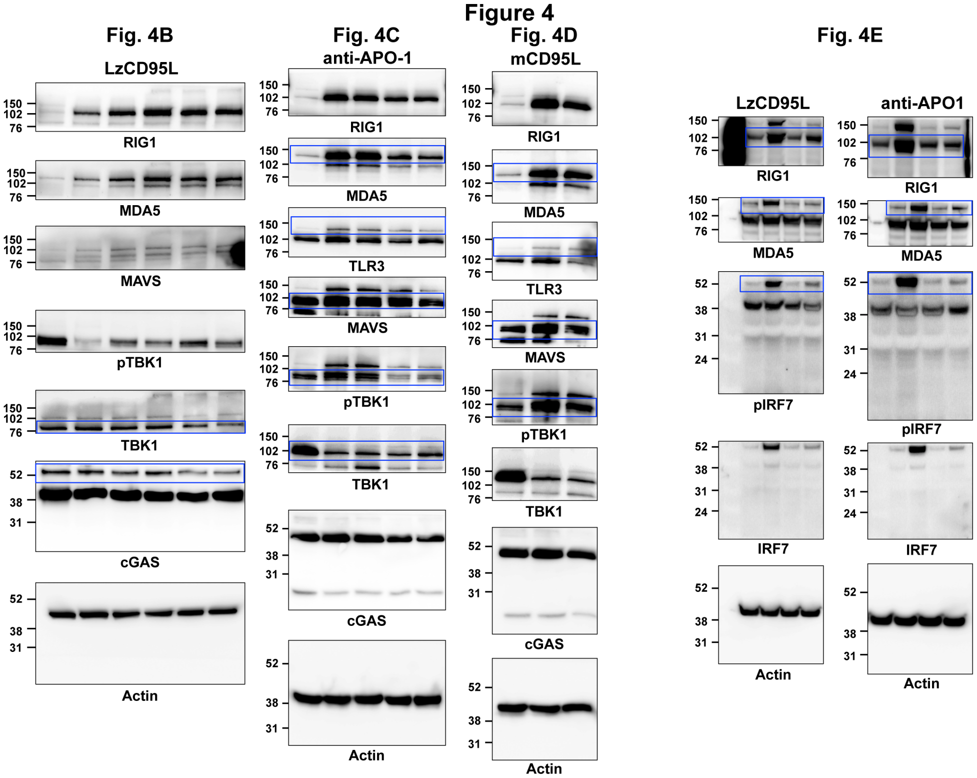

Figure 4

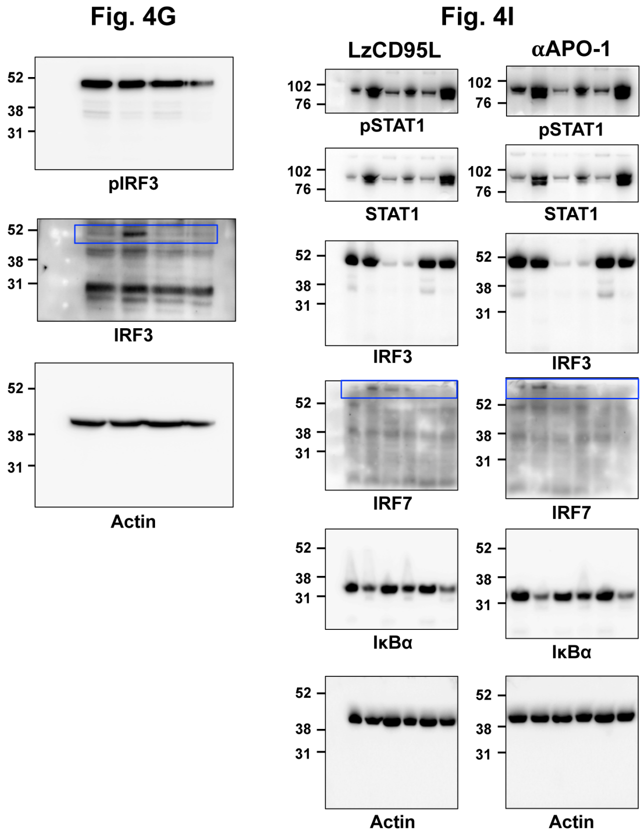

Figure 6

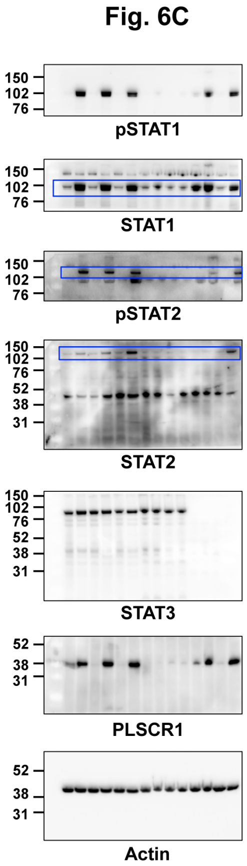

Figure S1

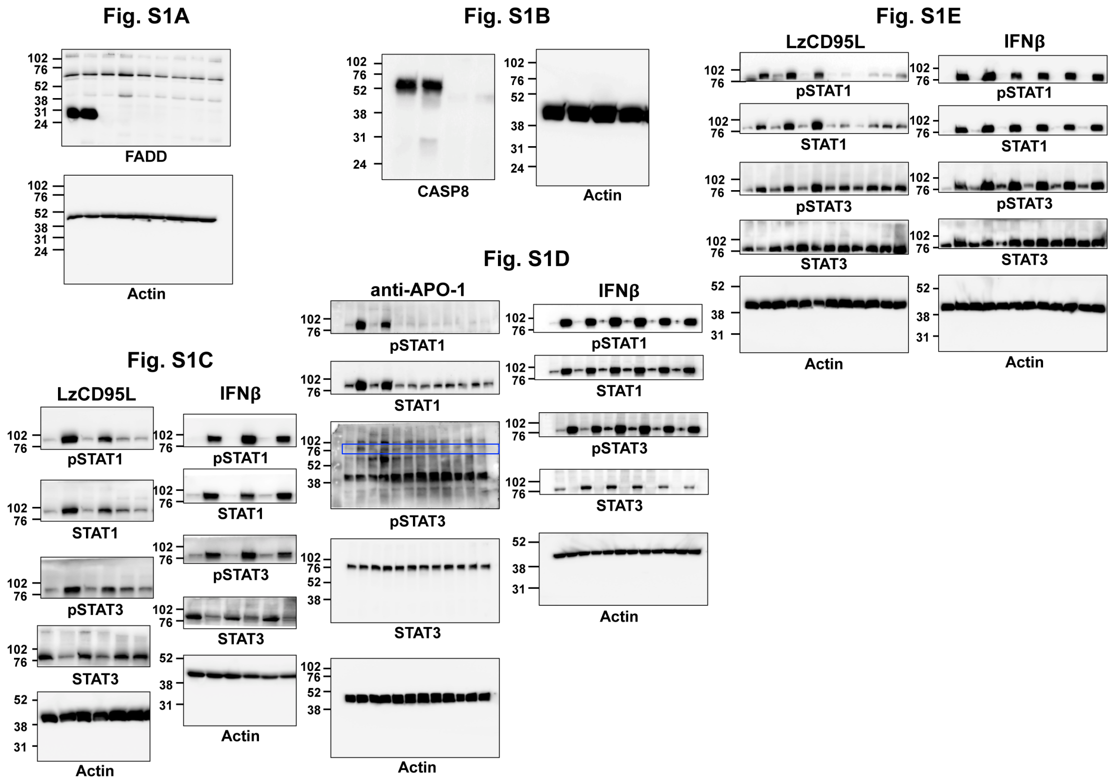

Figure S3

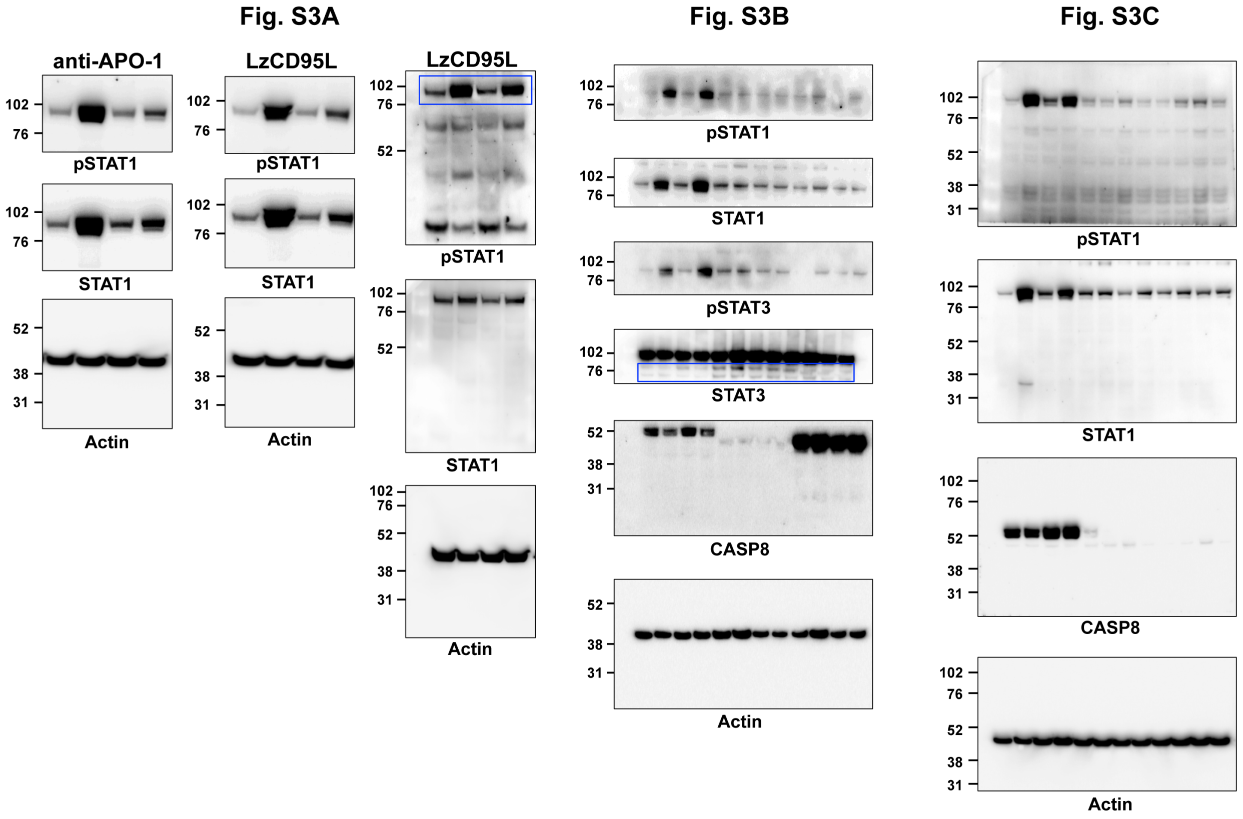

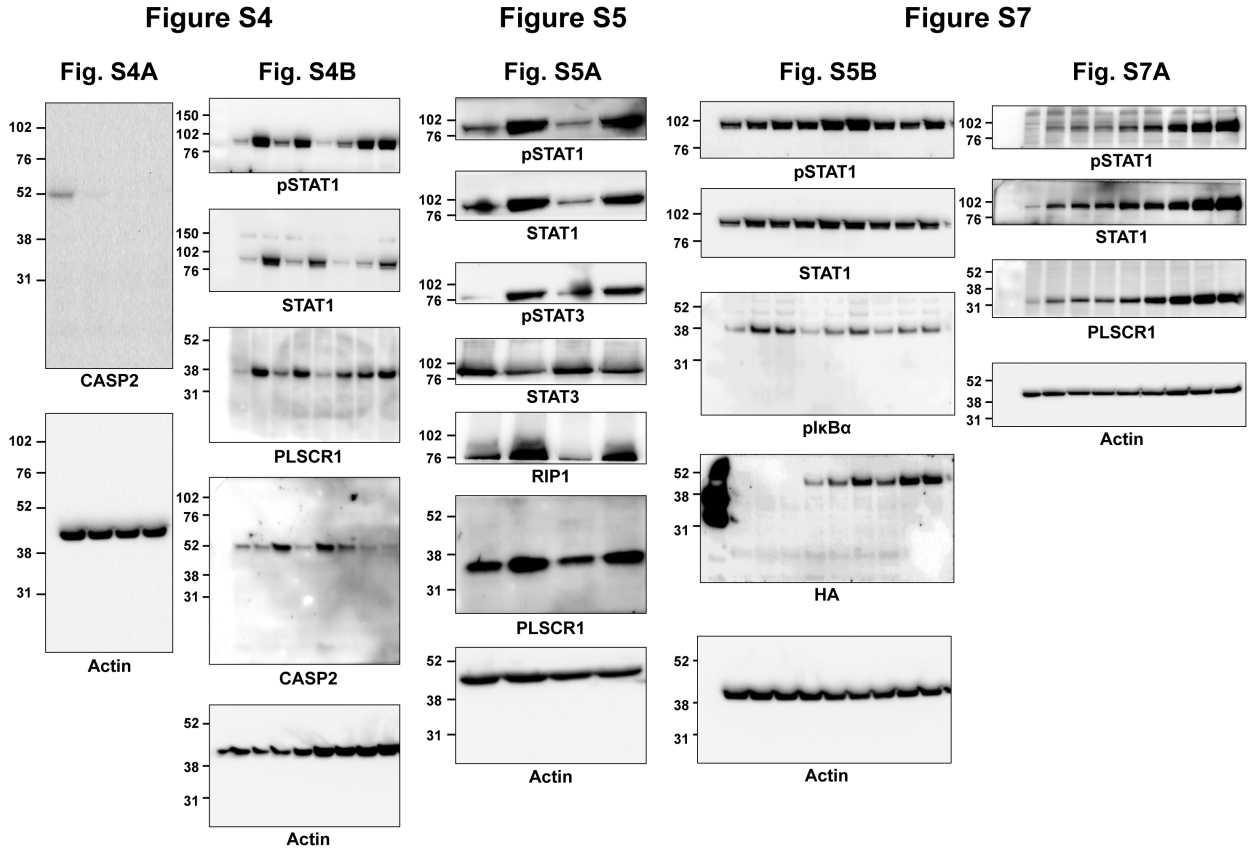

Figure S9. All uncropped immunoblot images. Molecular weights are given for each blot in kDa. Regions used in the figures are boxed in blue when unclear.
